# Bone Histology in *Dysalotosaurus lettowvorbecki* (Ornithischia: Iguanodontia) – Variation, Growth, and Implications

**DOI:** 10.1371/journal.pone.0029958

**Published:** 2012-01-06

**Authors:** Tom R. Hübner

**Affiliations:** Niedersächsisches Landesmuseum Hannover, Hannover, Germany; Ecole Normale Supérieure de Lyon, France

## Abstract

**Background:**

*Dysalotosaurus lettowvorbecki* is a small ornithopod dinosaur known from thousands of bones and several ontogenetic stages. It was found in a single locality within the Tendaguru Formation of southeastern Tanzania, possibly representing a single herd. *Dysalotosaurus* provides an excellent case study for examining variation in bone microstructure and life history and helps to unravel the still mysterious growth pattern of small ornithopods.

**Methodology/Principal Findings:**

Five different skeletal elements were sampled, revealing microstructural variation between individuals, skeletal elements, cross sectional units, and ontogenetic stages. The bone wall consists of fibrolamellar bone with strong variability in vascularization and development of growth cycles. Larger bones with a high degree of utilization have high relative growth rates and seldom annuli/LAGs, whereas small and less intensively used bones have lower growth rates and a higher number of these resting lines. Due to the scarcity of annuli/LAGs, the reconstruction of the life history of *Dysalotosaurus* was carried out using regularly developed and alternating slow and fast growing zones. *Dysalotosaurus* was a precocial dinosaur, which experienced sexual maturity at ten years, had an indeterminate growth pattern, and maximum growth rates comparable to a large kangaroo.

**Conclusions/Significance:**

The variation in the bone histology of *Dysalotosaurus* demonstrates the influence of size, utilization, and shape of bones on relative growth rates. Annuli/LAGs are not the only type of annual growth cycles that can be used to reconstruct the life history of fossil vertebrates, but the degree of development of these lines may be of importance for the reconstruction of paleobehavior. The regular development of annuli/LAGs in subadults and adults of large ornithopods therefore reflects higher seasonal stress due to higher food demands, migration, and altricial breeding behavior. Small ornithopods often lack regularly developed annuli/LAGs due to lower food demands, no need for migration, and precocial behavior.

## Introduction

### In General

Ever since scientists began to work with the remains of those extinct animals that lack direct living descendants, they dreamed of being able to accurately reconstruct life histories and, at least partially, social structures and behavior. Unfortunately, it is almost impossible to obtain such fundamental information using only morphological and/or statistical methods, because absolute ontogenetic dates of age or time of sexual maturity are not determinable. Size classes within a bonebed of a single species, surface texture of bones, or degree of suture closure are examples of tools often used to estimate relative age and ontogenetic status of fossil animals, but these methods are always highly imprecise (e.g. [1]–[4]). The study of bone histology has enabled paleontologists partially filling this methodological gap, because its insights can provide the required absolute data in many cases (see e.g. [5]–[10] for a general introduction into bone histology and common terms).

The basal iguanodontian ornithopod dinosaur *Dysalotosaurus lettowvorbecki* was the subject of this study. *Dysalotosaurus* was found during the famous German Tendaguru expeditions of 1909 to 1913, which took place 60 km west of the seaport of Lindi, southeast Tanzania [11], [12]. In contrast to the abundant remains of sauropods and the stegosaur *Kentrosaurus*, *Dysalotosaurus* is known from only a single locality, but the two closely related monodominant bonebeds found in channel lag deposits [13] produced thousands of bones of a minimum number of 100 individuals, from several growth stages, and in all degrees of disarticulation [9]. Although the genesis of this mass accumulation has long been discussed as of either catastrophic or attritional origin [14]–[16], the available taphonomic record currently favors the catastrophic mortality of a single herd [9]. Preburial weathering and signs of scavenging (widely distributed bones, tooth marks, a significant number of shed carnivore teeth) are absent, which implies fast burial after death. Abrasion is also unknown and there is only slight sorting of bones in favor of large and/or robust elements. The bonebeds are therefore autochthonous or parautochthonous in origin. A preservational difference between the two bonebeds, the upper of which almost overlies the lower, is not recognizable. Thus, a single *Dysalotosaurus* herd was probably trapped in one of the numerous tidal channels of that ancient coastal plain [17], drowned in a spring tide, and their graveyard was reworked at least once by another spring tide (a process that can take place every two weeks) resulting in the split into two separate bonebeds. A more detailed analysis of the taphonomy of the *Dysalotosaurus* quarry will be published in a subsequent paper.

An ontogenetic series of femora of *Dysalotosaurus* was previously studied by Anusuya Chinsamy-Turan [18] under the name *Dryosaurus lettowvorbecki*. The generic name *Dysalotosaurus* was made a synonym of *Dryosaurus* by Galton [19] due to many morphological similarities between *D. lettowvorbecki* and *D. altus*. However, an ongoing revision of the anatomy of both taxa (see also [9] and [20] for comments) revealed numerous significant anatomical differences in several parts of the skeleton, which clearly support the resurrection of the genus *Dysalotosaurus*.

### Age Estimations via Bone Histology

In many recent tetrapods, one growth cycle commonly represents one year of time (e.g. [5], [6], [8], [10], [21], [22], [23]), and this observation has been commonly used to estimate age for extinct tetrapods (e.g. [24]–[30]). This fact is the basis of the method of skeletochronology [31].

However, an accurate count of the number of annuli and/or LAGs (Lines of Arrested Growth) is often hampered by the ontogenetic expansion of the marrow cavity and/or secondary remodeling. This problem was often solved by the back-calculation of the lost/obscured number of annuli/LAGs [5], [28], [32], [33], [34]) or by the examination of an ontogenetic series (e.g. [27], [35], [36]).

There is also a high variability in the number of annuli/LAGs between different individuals within a single population (e.g. [22]), between different skeletal elements of one individual (e.g. [33], [36], and sometimes even in the cross section of a single bone (e.g. [37]). For example, single individuals of the dinosaurs *Plateosaurus* [32], [33], *Maiasaura* [36], and *Hypacrosaurus* [38] show different numbers of preserved LAGs in different skeletal elements, depending upon the general anatomical condition and specific growth pattern of each of these elements (e.g. cortical thickness, growth rate, rate of remodeling etc.).

A last important point is the assumption that all annuli/LAGs counted in a bone are indeed true annual layers. These lines can also be generated as a result of environmental stress, such as scarcity of food, illness, or during seasons of pairing or reproduction [5]. It is also possible to find double LAGs, which are consistently close together and represent a single year. Some tropical mammals, for instance, can even generate two cycles per year [22]. All these deviations from the simple annual model of growth cycles are rarely discernable in extinct species (e.g. [33]) and must be treated as sources of error in the calculation of individual age.

Another actualistic method used to estimate relative age of extinct animals is ‘Amprino's Rule’ (e.g. [32]). Amprino [39] suggested that similar bone tissues in different animals reflect similar growth rates. It is now widely accepted that maximum body size seems to be one of the major factors that influences growth rates, and therefore indirectly influences bone tissue types [40]–[44]. There are also differences in growth rate between different elements within a single skeleton (e.g. [32], [36], [40], [45]) and during ontogeny (e.g. [5], [18], [34], [38]). However, recent studies of birds and reptiles recognized a clear correlation between growth rate and the size and density of vascular canals, but no correlation between growth rate and orientation of vascular canals [40], [41], [45], [46]. Such a correlation seems to exist only due to extreme environmental conditions, which force an animal to generate extraordinarily high growth rates [47]. Thus, ‘Amprino's Rule’ can help to estimate the growth rate of an extinct species, but, as for skeletochronology, the results are strongly dependent on body size, ontogenetic stage, and skeletal element and should always be considered in comparison with other individuals, populations, and species.

### Bone Histology in Ornithopod Dinosaurs

Ornithopods are one of the best studied dinosaur groups with regard to bone histology, because several taxa are known from many individuals of different growth stages [18], [34], [36], [38], [48]–[55]. It has even proved possible to reconstruct the breeding strategy (altricial or precocial) and life history for some taxa. However, whereas the growth pattern of large ornithopods is quite well understood, the bone histology of many small ornithopods has raised more questions than answers as to their growth patterns [6], [18], [48], [52], [55]. In particular, the scarcity or even absence of annuli/LAGs, the usual tool for age estimations, has considerably complicated the reconstruction of their life history. The recent discovery that annuli/LAGs are indeed present in *Dysalotosaurus* and its close relative *Dryosaurus* ([9], [52], in contrast to [18]) helped in interpretations of their growth patterns. However, the inconsistent development of annuli/LAGs made it necessary to examine another type of growth cycle for the reconstruction of the life history of *Dysalotosaurus* [9]. Additional types of possible annual markers were previously documented mainly in sauropods (e.g. [56], [57], [58]). The annual development of the type of growth cycles used here has been assumed previously [59], but the application of these growth cycles in order to reconstruct life history is successfully made here for the first time.

Observations of bone tissue types as well as vascular and fibrillar organization in different skeletal elements of *Dysalotosaurus* led to some important insights into the reasons behind these multiple variations. Furthermore, the highly inconsistent development of annuli/LAGs and the newly described type of annual growth cycles resulted in a new hypothesis to explain the differences in growth patterns between large and small ornithopods.

## Results

The description of the microstructure of the sampled bones will be restricted to the main features of cross sectional shape, vascularization, and development of growth cycles. Where appropriate, the microstructure of the femur will also be compared to the description provided by Chinsamy [18]. A complete version of the description summarized here is available in the supporting material (Text S1).

In sum, 70 individual bones were sampled comprising 30 femora, 12 tibiae, 13 humeri, seven fibulae, and eight prepubic processes, but not all of them could be used for quantitative analyses due to insufficient preservation.

### Bone Histology of the Femur of *Dysalotosaurus*

#### Description

**Figure 1:**
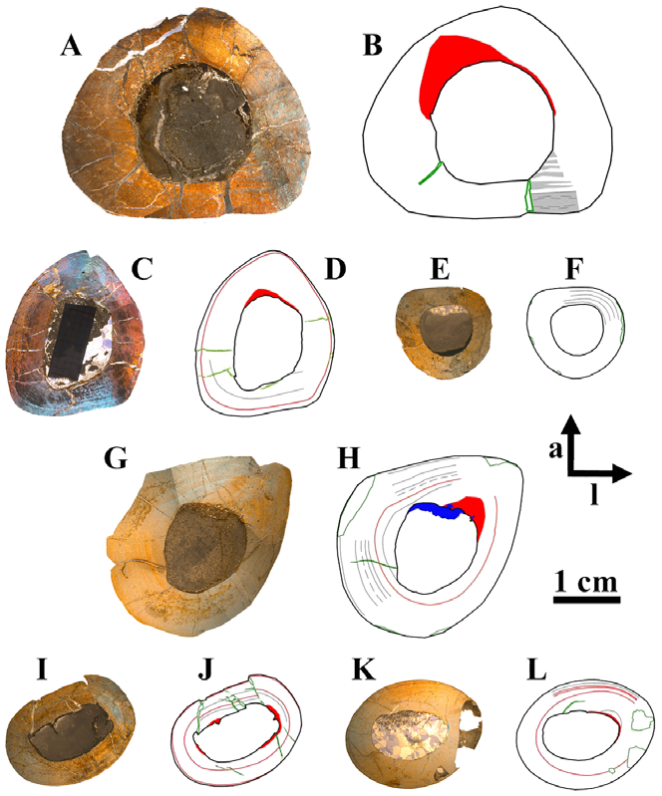
Representative cross sections and corresponding sketches of femora, tibiae, and humeri. A–B: Large femur GPIT/RE/3588, cut distally to the base of the fourth trochanter; C–D: Medium-sized femur GPIT/RE/3587, cut close to the base of the fourth trochanter; E–F: Medium-sized tibia GPIT/RE/3724, cut proximal to the lateral bulge; G–H: Large tibia SMNS T 3, cut close to the lateral bulge; I–J: Large humerus GZG.V 6223, cut distal to the mid diaphysis; K–L: Large humerus GPIT/RE/4877/8929, cut proximal to the mid diaphysis. All sections are oriented and scaled consistently. Internal red area represents CCCB (B, D, H, J) or an endosteal layer (L). Lines in green mark cross sectional damage. Growth cycles are shaded (B) or lined (D, F, H, J, L) in gray, annuli/LAGs are lined in red. The blue area in H represents medullary bone.

The femoral cross section is generally triangular in shape and becomes more slender close to the base of the fourth trochanter (see Fig. 1A–D for the general orientation). The respective cross sections of figure 1 in Chinsamy [18] are inconsistently oriented, so that the larger section (from a left femur) is oriented with its anteromedial wall facing ventrally and the smaller section (from a right femur) is oriented with its posteromedial corner in that way.

**Figure 2 pone-0029958-g002:**
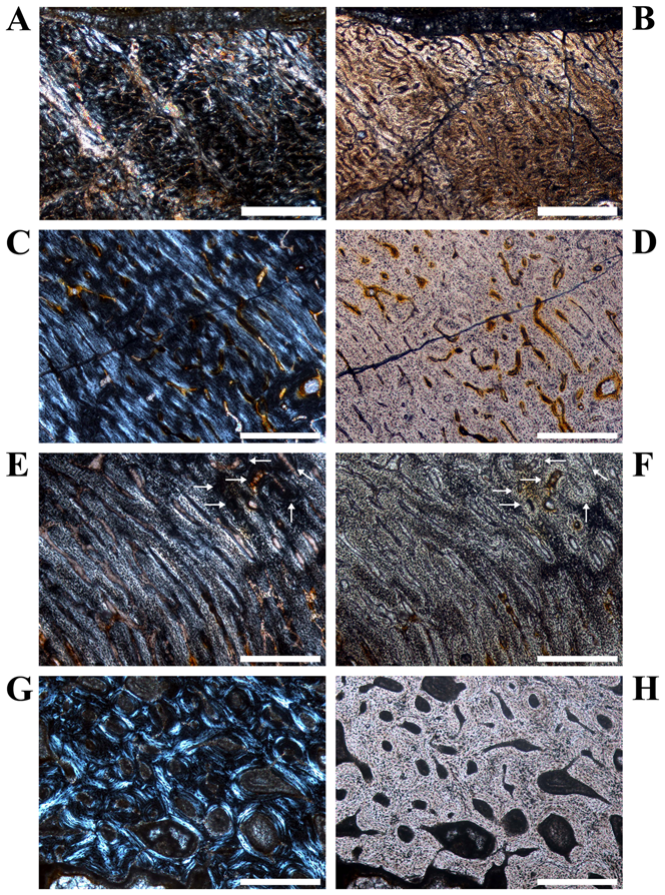
Details of cross sections of *Dysalotosaurus* femora showing resorption and bone tissue types. A–B: GPIT/RE/3588, A – Interior margin of posterolateral corner demonstrating the resorptive nature of the marrow cavity, under polarized light. Note the angle of the zonation towards the marrow cavity at the top. B – The same as in A under normal light. C–D: SMNS F 2, C – Part of the posterolateral bone wall with numerous primary osteons under polarized light. The orientation of bone fibers and primary osteons varies between the darker and the strongly birefringent zones. The marrow cavity lies in the direction to the top right. D – The same as in C under normal light. E–F: GPIT/RE/3587, E – Part of the anterior corner under polarized light showing parallel-fibered tissue with mainly circumferential primary osteons and a small cluster of secondary osteons at the top right (arrows). F – The same as in E under normal light. G–H: GPIT/RE/3414, G – Part of the anterior wedge of CCCB (Compacted Coarse Cancellous Bone) under polarized light. Note the difference between the continuous transitions between the trabeculae of the CCCB and the interruptions in the lamellar bone originating from secondary osteons of various developmental stages. The marrow cavity is located at the bottom. H – The same as in G under normal light. Scale bars = 1 mm in A, B. Scale bars = 500 µm in C–H.

The edge of the marrow cavity is well defined and mainly consistent, but undulations and cavities are often present internal to the anterior corner. No spongiosa were observed within the marrow cavity. A layer of endosteally deposited lamellar bone may be developed in variable thicknesses around the marrow cavity, although it never forms a completely surrounding band. One reason is the resorptive posterior edge of the cavity (e.g. Figs. 1B, D; 2A–B).

The compact bone wall consists mainly of two types of bone tissue. Most of it is composed of periosteal fibrolamellar bone tissue with woven fibered matrix and numerous primary osteons (Fig. 2A–D). Only the anterior corner shows sometimes strongly birefringent parallel-fibered matrix (Fig. 2E–F) and is most likely the same region mentioned for a large femur by Chinsamy [18]. The second tissue type, compacted coarse cancellous bone (CCCB), is of endosteal origin and mostly restricted to the anterior corner and adjacent areas (marked in red in the sketches of Fig. 1B, D; 2G–H). In more distal sections, the amount of CCCB relative to fibrolamellar bone, and the average size of the innermost canals of CCCB, increases.

**Figure 3 pone-0029958-g003:**
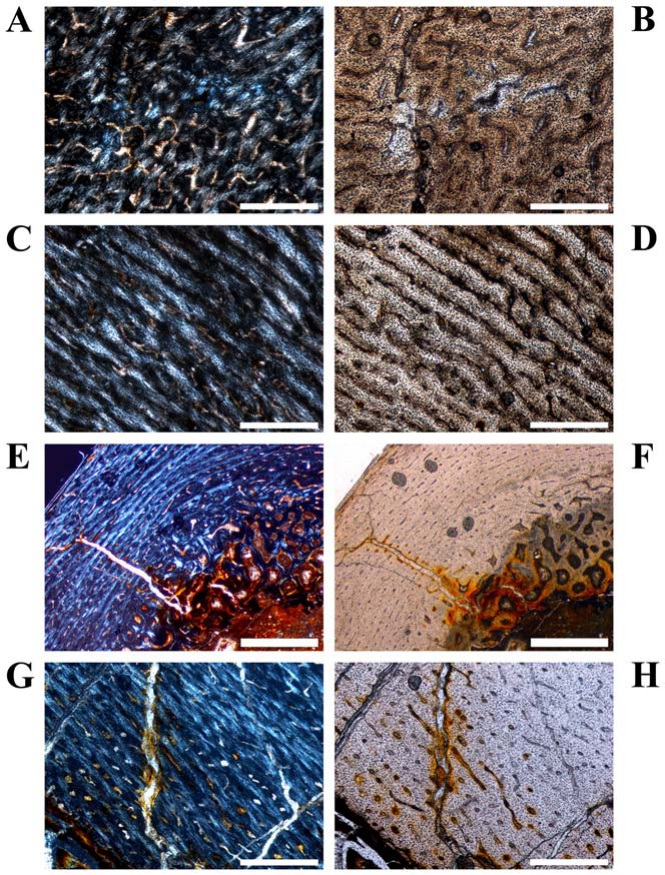
Variation of vascular organization in cross sections of *Dysalotosaurus* femora. A–D: GPIT/RE/3588, A – Part of the medial corner with numerous poorly organized primary osteons and weakly birefringent bone matrix, under polarized light. B – The same as in A under normal light. C – Part of the lateral wall with well organized laminar and circumferential primary osteons as well as mainly transverse and strongly birefringent bone fibers, under polarized light. D – The same as in C under normal light. E–F: GZG.V 6590 28, E – Medial part of the anterior corner with the CCCB wedge involving about half of the bone wall thickness and apparently compressing the primary bone wall exteriorly, under polarized light. F – The same as in E under normal light. G–H: SMNS F2, G – Internal part of the anterior corner beside the CCCB wedge (starts beyond the left frame of the image) showing the typical knitted pattern with small, laminar and mainly longitudinally oriented primary osteons nested between thick cords of matrix, under polarized light. The marrow cavity is at the bottom to the left. H – The same as in G under normal light. Scale bars = 500 µm in A–D, G–H. Scale bars = 1 mm in E–F.

The vascularization (*sensu lato*, following [6]) is very variable in terms of the size of the canals and overall density. Most of the vascular canals are well-developed primary osteons. Generally, the size and density are greatest in the thickest parts of the primary bone wall (posteromedial corner, Fig. 3A–B) and lowest, with relatively more matrix between the primary osteons, in the thinnest parts (Fig. 3C–D). The latter also include the anterior corner of the femur, because the CCCB wedge takes up the inner part of the bone wall in this area and the outer primary bone looks compressed (Fig. 3E–F). The opposite relationship exists for the degree of organization of vascular canals, where it is highest in the thinner parts of the primary bone wall (longitudinal to laminar orientations) and very low in the thickest parts (plexiform to sometimes reticular orientations; compare Figs. 3C–D with 3A–B). An additional tendency is the general increase of vascular organization from inner parts of the bone wall towards the periosteal surface. However, the laminar type of vascularization is the most abundant. The smallest, longitudinal, and fairly well-organized primary osteons are observable in the innermost areas of the primary bone wall around the anterior corner. There are relatively thick bands of matrix, which isolate these osteons from each other and which resemble a knitted pattern (Figs. 3E–H).

**Figure 4 pone-0029958-g004:**
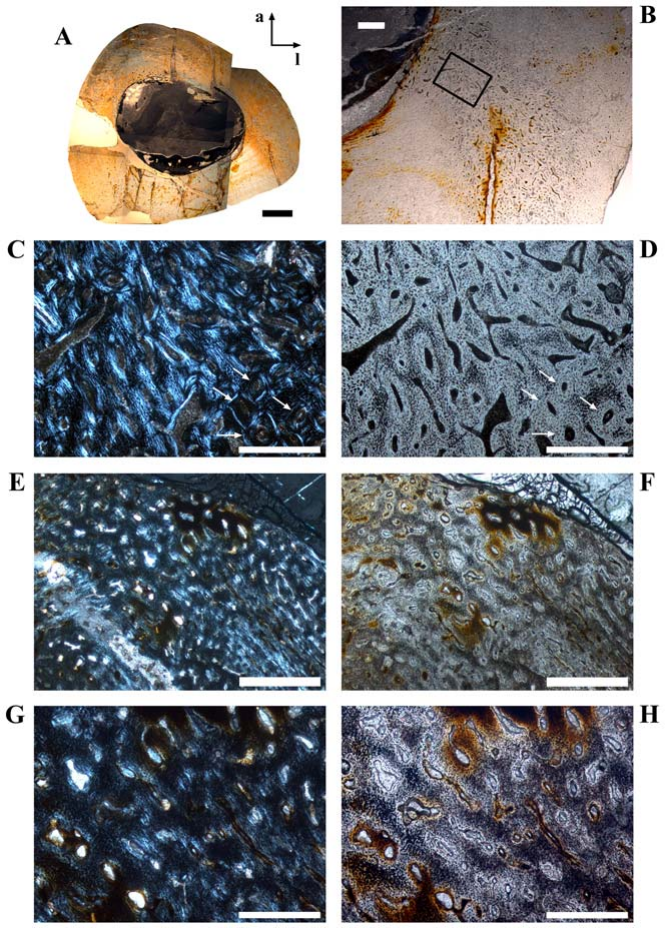
Cross sectional units in *Dysalotosaurus* femora with unusual bone tissue. A–H: GPIT/RE/3414, A – Overview of the oriented cross section (a = anterior, l = lateral), under normal light. B – Magnification of the lateral corner demonstrating the interruption of the usual vascularisation by the cloud of reticular canals of the Posterolateral Plug, under normal light. C – Magnification of the section framed in B, under polarized light. Arrows indicate scattered secondary osteons. D – The same as in C under normal light. Note the high density of osteocyte lacunae. E – Magnification of the anterior corner under polarized light and slightly rotated clockwise relative to A. F – The same as in E under normal light. G – Magnification of the upper center of E. Note the weak development of many primary osteons. H – The same as in G under normal light. Scale bars = 5 mm in A. Scale bars = 1 mm in B, E–F. Scale bars = 500 µm in C–D, G–H.

The posterolateral corner represents a special area of the bone wall (Fig. 4A–B). Here, primary osteons are less well developed, larger on average, and more randomly shaped and oriented than in all other cross sectional units (Fig. 4C–D). This area, which will be called the Posterolateral Plug in the following text, represents a very abrupt change within the organization of bone tissue. The general course of growth cycles, bone laminae, and the orientation of vascular canals stops at the border of the Posterolateral Plug (Fig. 4B) and only distinct annuli/LAGs can be followed through it. This area is most prominent in sections slightly distal to the midshaft and becomes less prominent proximally, towards the fourth trochanter. A similar structure is sometimes visible in the outer cortex of the anterior corner of more proximal sections, and in larger sections (Fig. 4E–H). This cluster, however, does not significantly disturb the general organization of the tissue and is also far less widespread.

**Figure 5 pone-0029958-g005:**
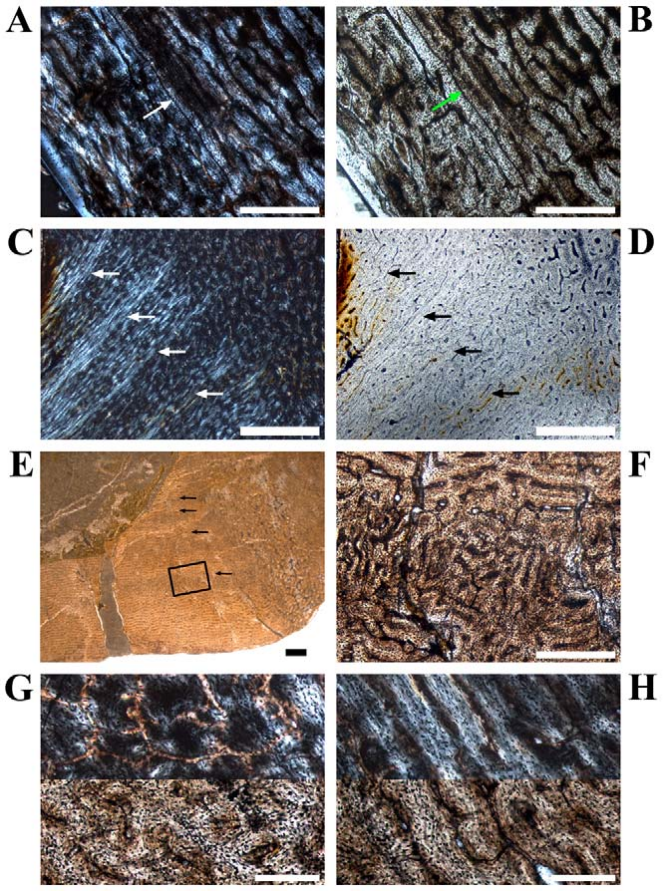
Growth cycles in *Dysalotosaurus* femora. A–B: GPIT/RE/3587, A – The outer edge of the posterior bone wall with mainly circumferential primary osteons and a LAG (arrow), under polarized light. B – The same as in A under normal light. C–D: GPIT/RE/3414, C – Interior part of the posterolateral bone wall under polarized light. The growth cycles (fast growing zones darker, slow growing zones brighter) stop at the Posterolateral Plug. Arrows indicate the outer edge of a slow growing zone. D – The same as in C under normal light. The growth cycles are now very difficult to identify. The best verifiable slow growing zones are the second and third where the outer edge is less vascularized and the circumferential orientation of canals is significant. E–H: GPIT/RE/3588, E – Posterolateral corner under normal light with the typical alternation of fast (darker) and slow (brighter) growing zones. The external edge of the slow growing zones is marked by arrows. Note the transition of the internal cyclicity to an almost uniform slow growing zone externally (border at the lower edge of the frame). F – Magnification of the section framed in E showing the strong organizational difference between primary osteons of the fast growing zone (center) and the slow growing zones (top and bottom). G – Close up of a fast growing zone both under polarized and normal light. The image is slightly rotated in comparison to E and F. H – Close up of a slow growing zone both under polarized and normal light. The image is slightly rotated in comparison to E and F. Scale bars = 1 mm in C–E. Scale bars = 500 µm in A–B, F. Scale bars = 200 µm in G–H.

The zonation pattern is also highly variable. Annuli/LAGs are present (in contrast to [18]; Fig. 5A–B), but only in 10 out of 30 sampled femora. There is additionally no correlation between the size of the bone and the number of annuli/LAGs (compare Fig. 1B, D). None of the cross sections record more than one or two annuli/LAGs. Nevertheless, these are the only growth cycles that can be followed around the cross section.

Another type of growth cycle is much more abundant, but less distinctive than annuli/LAGs because it is often only clearly visible under polarized light (Fig. 5C–D). This type is most developed within the lateral side of the posterior wall close to the Posterolateral Plug (Figs. 1A–D; 5E). It consists of weakly birefringent fast growing zones (viewed under polarized light) with mainly longitudinally oriented collagen fibrils, as well as numerous and dense primary osteons that show a relatively lesser degree of organization (Fig. 5F–G). The fast growing zones alternate with more strongly birefringent slow growing zones, which consist of mainly transversely oriented collagen fibrils and less dense and more circumferentially oriented primary osteons that show a relatively higher degree of organization (Fig. 5F, H). The transition from the fast to the slow growing zone is diffuse. Only the external rim of the slow growing zones is definable and possible annuli/LAGs occur mainly in this area. Thus, one growth cycle consists of an internal fast growing zone and an external slow growing zone. The slow growing zones often merge together in the thin parts of the primary bone wall (especially anteriorly) or split up towards thicker parts, where they even vanish in some areas. One has therefore to check carefully their number and extension by repeatedly rotating the cross sections under polarized light. The Posterolateral Plug interrupts the course of these growth cycles completely (Figs. 4A; 5C–E).

**Figure 6:**
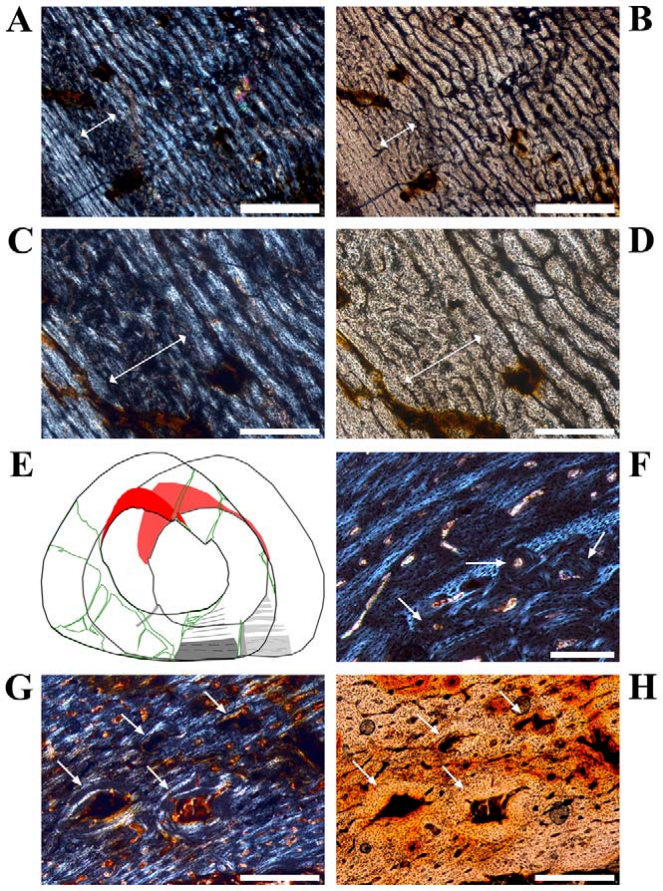
The Mark of Initial Sexual Maturity (MISM) as well as interior details of the anterior corner in large sampled femora of *Dysalotosaurus*. A–D: SMNS F1, A – Part of the posterior bone wall, under polarized light, with the most external fast growing zone (double-headed arrow) and the transition to the thick, non-cyclical slow growing area externally (centre and right of the image). This transition is the MISM. B – The same as in A under normal light. C – Magnification of the top left of A under polarized light. The MISM is again at the right end of the double-headed arrow. Note that the MISM is not a sharp line but just another transition from fast to slower growth without any further fast growing zones towards the periphery. D – The same as in C under normal light. E: GPIT/RE/3414 (in front) and GPIT/RE/3588 (in the back), the sketches demonstrate the perfect overlap of the zonation as well as the MISM in both large femora. The slow growing zones are shaded in the back and their external rim is marked in the front. The dashed lines within the thick external slow growing zone (shaded in both representing growth after reaching sexual maturity) mark unsecured growth cycles. F: GZG.V 6590 28, Close up of the border between the CCCB wedge internally (bottom right) and the primary bone tissue externally within the anterior corner. Secondary osteons are marked by arrows. Note the knitted pattern of the primary bone tissue. G–H: GZG.V 6211 22, G – Internal part of the anterior corner close to the CCCB wedge (starts at the lower right) with knitted pattern of the bone tissue internally and some scattered secondary osteons (arrows) still under development, under polarized light. H – The same as in G under normal light. Scale bars = 1 mm in A–B. Scale bars = 500 µm in C–D, G–H. Scale bars = 200 µm in F.

Five out of six of the largest sectioned femora show a transition (Mark of Initial Sexual Maturity – MISM, see below) from the generally distinct sequence of growth cycles internally to a much more uniform area externally. The latter resembles a very thick slow growing zone and only a very weak internal zonation is recognizable (Figs. 1A–B; 5E; 6A–E).

Secondary remodeling is very rare, which differs from the remarks of Chinsamy [18]. There are only local occurrences of scattered secondary osteons, concentrated mainly in the transitional area between the primary bone tissue and the CCCB (Fig. 6F–H). Isolated osteons are also present within the latter (Fig. 2G–H). Other isolated occurrences are located within the Posterolateral Plug (Fig. 4C–D) and sometimes in the external part of the anterior corner (Fig. 2E–F). However, sections from more distal parts of the femur have greater numbers of secondary osteons throughout the cortex.

**Figure 7:**
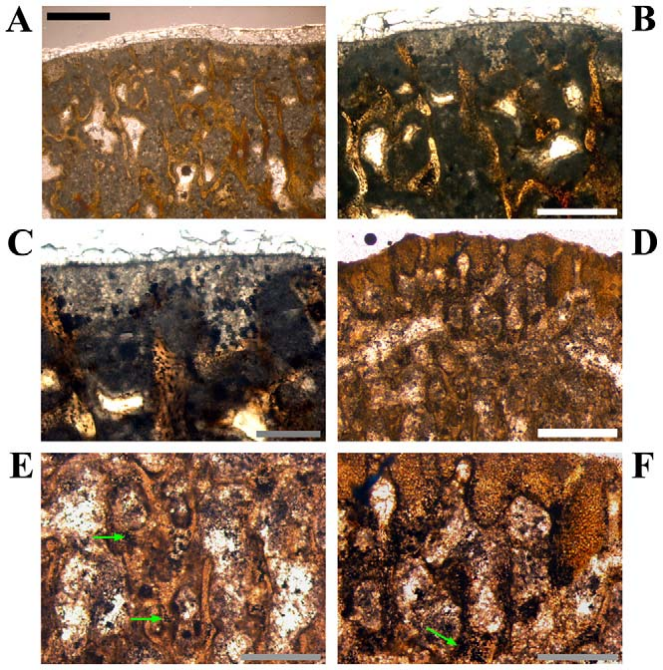
Upside down images of longitudinal sections of the distal ends of two femora. A–C: large specimen GPIT/RE/3518, A – Overview under normal light. The foam-like patches at the distal (here upper) edge consist of calcified cartilage partially divided by trabecular bone. B – Magnification of the upper centre of A under normal light. C – Magnification of the upper centre of B under normal light. The bubbles of calcified cartilage cells are well distinguishable from the osteocyte lacunae within the trabecular bone. D–F: Small specimen GZG.V 6379, D – Overview under normal light. The pads of calcified cartilage reach deeper into the specimen than in A. Trabecular bone is well ossified in the lower centre but there are already centres of ossification close to the distal (here upper) surface. E – Magnification of the lower centre of D. Isolated clusters of calcified cartilage are still present (arrows). F – Magnification of the upper centre of D showing trabecular bone under development and isolated remains of calcified cartilage within bone (arrow). Scale bars = 1 mm in A, D. Scale bars = 500 µm in B, E–F. Scale bars = 200 µm in C.

The comparison of longitudinal sections of a large femur and of the smallest sampled femur (Fig. 7) reveals that the amount and area occupied by pads of calcified cartilage decreases with size, but there is still a substantial amount present in the large specimen. The large specimen is much better ossified than the small specimen, consisting of a dense meshwork of trabecular bone. However, there is a concentration of bony straps in the epiphyseal centre of the small specimen, which reaches almost to the distal end.

### Ontogenetic Stages in Femora

Due to the highly variable features within the shaft, between different femoral cross sections, and even within a single section, ontogenetic stages are difficult to distinguish. The use of most of the features, such as the degree of development of primary osteons, vascularization pattern, or secondary remodeling, was therefore limited, and there is often a smooth transition between successive ontogenetic stages. However, useful indicators of ontogenetic stage are, in addition to absolute size, the number of growth cycles and the degree of development of distinct areas, such as the Posterolateral Plug.

#### Stage 1 or Embryonic/Perinatal Stage

This stage, already described in some other ornithopods [36], [51], [52], is not represented in the sampled femora of *Dysalotosaurus*, and the overall size of other known specimens indicates that none of the preserved femora would fit into this stage.

#### Stage 2 or Early Juvenile Stage (Fig. 8A–D; Tab. 1)

**Figure 8:**
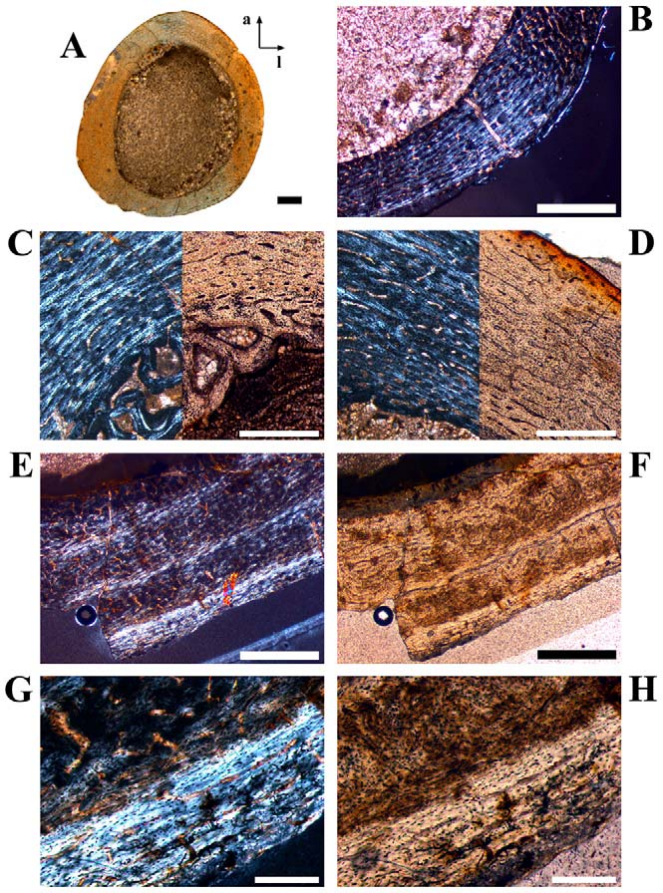
Bone histology and growth cycles in juvenile femora of *Dysalotosaurus*. A–D: GZG.V 6379, A – Orientated overview (a = anterior, l = lateral) under normal light. Note the wide marrow cavity compared to the bone wall thickness in this early juvenile specimen. B – Magnification of the posterolateral corner under polarized light with only a weak indication of the Posterolateral Plug. The knitted pattern of the bone tissue with mainly longitudinal primary osteons is dominant. C – Magnification of the interior of the anterior corner medially, under both polarized and normal light, with a CCCB wedge under development and the typical knitted pattern of the primary bone tissue. D - Magnification of the interior of the anterior corner laterally, under both polarized and normal light, with the typical knitted pattern of the primary bone tissue. The vascular pattern changes already to more circumferential primary osteons towards the periphery. E–H: GZG.V 6590, E – Three slow growing zones are well visible under polarized light. The Posterolateral Plug starts at the left edge of the image. F – The same as E under normal light. G – Magnification of the utmost slow growing zone with an annulus at its interior border. H – The same as in G under normal light. Scale bars = 1 mm in A–B, E–F. Scale bars = 500 µm in C–D. Scale bars = 200 µm in G–H.

**Table 1:** Basic dataset of all cross sections of femora used for correlations and the calculation of growth curves.

Labels	DMW	CM	Group	Ant-post Ø	Med-lat Ø	Ant-post cavity	Med-lat cavity	BWT max	BWT min	Number growth cycles	Number LAGs/annuli	Ontogenetic stage	Age in years
GZG.V 6379	16.1	29.1	2	8.9	7.8	6.3	5.2	1.8 medial	―	1?	―	early juvenile	<1
GZG.V 6653	24.9	42.4	3	16.6	11.8	8.8	6.9	5.2 posteromedial	1.6 posterolateral	2–3	―	late juvenile	2.15
GZG.V 6467	29.3	49	1	―	―	11	10.8	3.9 medial	1.8 posterior	2–3	―	late juvenile	3.58
SMNS F 14	30.6	51	2	16.3	15.1	11.1	9.2	3.5 medial	―	3	―	late juvenile	3.98
GPIT/RE/5650	32.3	53.4	1	―	―	11.1	9.4	―	―	2	―	late juvenile	4.45
GZG.V 6665	33.3	55.2	3	19.2	17.2	8.9	9.2	7.1 posteromedial	3 posterior	2	―	late juvenile	4.79
GZG.V 6652 26	33.3	55	2	17.4	14.6	11.3	8.9	4.1+ medial	2.1 posterior & lateral	2	―	late juvenile	4.75
GZG.V 6590	31.6	53	2	17.3	13.3	10.4	7.8	4.3 posteromedial	2.1 posterolateral	3	1	late juvenile	4.37
GZG.V 6590 28	35.3	58	1	18.5	18.1	11.8	11.7	4.1 medial	2.2 anterolateral	3	―	late juvenile	5.31
GZG.V 6386	35.3	58	1	20.2	16.2	12.7	9.9	4.9 posteromedial	2.3 posterolateral	3	―	late juvenile	5.31
GZG.V 6211 22	41.9	68	2	―	―	14.7	11.2	―	2.3 posterior	3–4	1	sex. immature	7.04
GPIT/RE/3587	44.6	72	2	26	20.7	14.9	10	6.8 anterior	4 posterior	4	1	sex. immature	7.69
GZG.V 6381/6434	45.9	74	2	23	21	13.4	10	6 posteromedial	3.8 posterior	4	1	sex. immature	8
SMNS F 4	52	83.2	1	23.5	25.4	14.6	15.1	5.9 medial	3.1 posterior	4	―	sex. immature to sex. mature	9.45
GZG.V 6395	70.4	111	1	―	34	20.5	14.9	12.5 posteromedial	―	7	1	sex. mature	14
GPIT/RE/3414	70.7	111.3	1	32.2	36.8	14.6	20.6	11.4 posteromedial	5.9 anterolateral	8	1	sex. mature	14.06
GPIT/RE/3588	72.5	114	1	33.3	37	18.7	18	11 medial	5 posterior	8	1	sex. mature	14.58
SMNS F 1	74.1	117.7	2	39.2	35.2	19.6	17.2	13.1 medial	5.5 posterolateral	7	―	sex. mature	15.36
SMNS F 2	77.8	122	2	38	37.2	20.1	19.1	10.8 medial	4.6 posterolateral	6	―	sex. mature	16.37

Explanation of heading-abbreviations: DMW – Distal mediolateral width; C – Midshaft circumference; Group – The group, into which the cross section was sorted, depends on cutting level; Ant-post Ø – Diameter of cross section in anteroposterior direction; Med-lat Ø – Diameter of cross section in mediolateral direction; Ant-post cavity – Diameter of marrow cavity in anteroposterior direction; Med-lat cavity – Diameter of marrow cavity in mediolateral direction; BWT max - Maximum of bone wall thickness; BWT min – Minimum of bone wall thickness. The age in years is derived from the growth curves. All data in mm.

The marrow cavity is very large compared to the bone wall thickness (see also [18]). The internal anterior wedge, if present, consists of CCCB that is not yet compacted. The posterolateral corner and the respective Plug are weakly pronounced. The periosteal compact bone tissue has a high number of longitudinal vascular canals. The primary osteons are often isolated from each other by thick bands of well-organized and relatively uniformly birefringent woven-fibered matrix (knitted texture; Fig. 8B–D). Particularly in the internal part anteriorly, only simple vascular canals are present. There is at most one slow growing zone developed at the external edge of the cortex (Fig. 8B).

#### Stage 3 or Late Juvenile Stage (Tab. 1)

The external circumferential profile is more pronounced and the Posterolateral Plug is well visible. The drift of the marrow cavity from approximately anterior to posterior is in progress, which is indicated by the well-compacted CCCB of a larger anterior wedge as well as a deeper incision into the posterior bone wall (this is also dependent on the sectioned level). The primary osteons are more numerous and there is a decrease in the proportion of knitted texture. There are the first occurrences of isolated secondary osteons. Growth cycles are well distinguishable and reach two to three in number (Fig. 8E–H).

#### Stage 4 or Sexually Immature Stage (Tab. 1)

The development of the external cross-sectional profile as well as of distinct areas (e.g. the Posterolateral Plug) is now complete (Fig. 1C–D). The anterior wedge of the CCCB is more pronounced, although this also depends on the relative position of the cross section within the shaft. The marrow cavity is deeply incised into the posterior wall (Fig. 1D). The density of well-developed primary osteons is very high in the thick and fast growing parts of the sections. Secondary osteons are more abundant and can also occur in the Posterolateral Plug and the anterior corner (Fig. 2E–F). The number of growth cycles is three to five.

#### Stage 5 or Sexually Mature Stage (Tab. 1)

The units of the cross sectional bone wall are strongly diversified (Fig. 1A–B). The anteroposterior migration of the marrow cavity interrupts up to four growth cycles posteriorly (Figs. 2A–B; 5E). Secondary osteons are numerous forming clusters anteriorly and posterolaterally at different distances from the external surface (Fig. 4C–D). The number of growth cycles reaches up to nine and the transition from well-distinguishable fast and slow growing zones internally to the diffuse and more uniform wide zone externally is visible in five of the largest cross sections (Figs. 1A–B; 4A; 5C–E; 6A–E).

### Bone Histology of the Tibia of *Dysalotosaurus*

#### Description

The cross-sectional shape of the tibia is almost egg-like in distal sections and almost circular in proximal sections, but there is always a straight anterior wall, which opposes the fibula when in articulation (Fig. 1E–H). The shape of the marrow cavity is more symmetrical than the external outline and the rim is mostly well defined and straight. A slight shift of the marrow cavity medially is observed in later ontogenetic stages.

**Figure 9:**
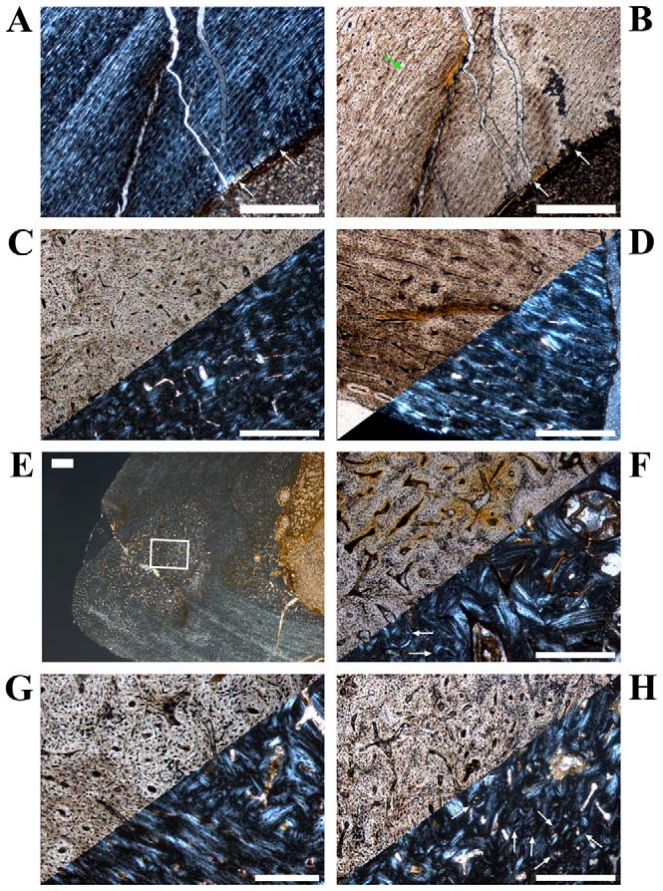
Vascular patterns and tissue types in a *Dysalotosaurus* tibia. A–H: Large tibia SMNS T3, A – Internal part of the lateral bone wall with laminar to sub-plexiform bone tissue under polarized light. Transversely oriented bone fibers dominate. The knitted pattern is visible at the right close to the marrow cavity. A thick endosteal layer is marked by white arrows. B – The same as in A under normal light. The external border of the prominent slow growing zone of A is also well visible here (green arrow). C – Strongly unordered primary osteons in a weakly birefringent woven matrix within the medioposterior corner under both polarized and normal light. D – Well organized primary osteons in a strongly birefringent almost parallel-fibered matrix at the outer edge of the lateral wall under both polarized and normal light. E – Overview of the anterolateral corner (here anterior to the bottom and lateral to the left) under polarized light. Note the whirl-like Anterolateral Plug within this corner, which interrupts the usual bone tissue, and the wedge of CCCB to the right at the marrow cavity. F – Partial close up of the CCCB wedge with the usual continuous lamellar bone and some interrupting secondary osteons (arrows), under both polarized and normal light. G – Close up of the border between CCCB (upper right) and primary bone tissue (lower left), under both polarized and normal light. The latter strongly resembles the juvenile knitted pattern. H – Magnification of the framed part in E showing an area within the Anterolateral Plug, under both polarized and normal light. Secondary osteons are marked with arrows. Scale bars = 1 mm in A–B, E. Scale bars = 500 µm in C–D, F, H. Scale bars = 200 µm in G.

An endosteal layer is developed almost exclusively in medium to large sections (Fig. 9A–B; see Tab. 2 for comparable sizes of samples) with its maximum thickness in the anteromedial or anterolateral corner. With one exception, the endosteal layer never completely surrounds the marrow cavity.

**Table 2 pone-0029958-t002:** Basic dataset of all cross sections of tibiae used for correlations.

Labels	DMW	Group	Ant-post Ø	Med-lat Ø	Ant-post cavity	Med-lat cavity	BWT max	BWT min	Number growth cycles	Number LAGs/annuli	Ontogenetic stage	Age in years
GPIT/RE/3795	17.3	1	6.3	―	4.6	4	1.3 anterolateral	0.9 anterior	―	―	early juvenile	<1
GZG.V 6434/6664	35.3	1/2*	12.2/12.8	14.5/12.9	6.8/5.9	7.2/5.8	4.4/4 anteromedial	2.1 lateral/2.8 anterior	2–4	―	late juvenile to sex. immature	3.1
GPIT/RE/5904	36.4	1	―	―	9.3	9.8	4.4 anteromedial	―	3	―	late juvenile to sex. immature	3.4
SMNS T 13	37.7	1	13.8	15	7.1	7.8	4.8 medial	2.1 lateral	2–4	―	late juvenile to sex. immature	3.8
GPIT/RE/4036	38	1	12.9	―	8.3	8.2	3.8 anterolateral	2.1 posterolateral	3	1	late juvenile to sex. immature	3.86
GPIT/RE/5755	38.1	2	13.3	12.6	7.2	6.3	3.8 anteromedial	2.7 anterior	3	―	late juvenile to sex. immature	3.91
GPIT/RE/3724	40.7	2	13.8	14.2	7.5	7.8	4 anterolateral	2.5 anterior	4	―	late juvenile to sex. immature	4.5
SMNS T 7	52.2	1/2*	13/12.8	―	9.2/10	9.1/―	4.8 show	―	3	2	late juvenile to sex. immature	7.5
SMNS T 3	75.7	1	25.3	27.6	13.2	12	10.7 anterolateral	4.4 posterolateral	7	1	sex. mature	15
GZG.V 6791	79.3	1	24	33	11.4	16.3	―	4.8 posterolateral	6	1	sex. mature	17

Explanation of heading-abbreviations: The headings are as in Table 1. The age in years was estimated by the comparison of relative positions within the size-frequency distributions of femora and tibiae, respectively.

*Each of these specimens provided a more proximal and a more distal sample, so that values for both cross sections were gained. All data in mm.

As in the femora, the tibial cross sections consist generally of fibrolamellar bone tissue with a high density of well-developed primary osteons, which are predominantly organized in a laminar pattern (Fig. 9A–B). The variability in size, density, and organization of vascular canals/primary osteons is also comparable to that seen in femora (Fig. 9). CCCB may occur as a wedge in the anterolateral corner internally, which extends far into the cortex only in the two largest cross sections (Figs. 1G–H; 9E–G). In most of the smaller sections (see below), as well as in the proximal sections, CCCB is absent. A structure similar to the femoral Posterolateral Plug is visible in the middle cortex of this corner (Fig. 9E, H), although its extent within the tibial shaft is much smaller than in the femur.

**Figure 10:**
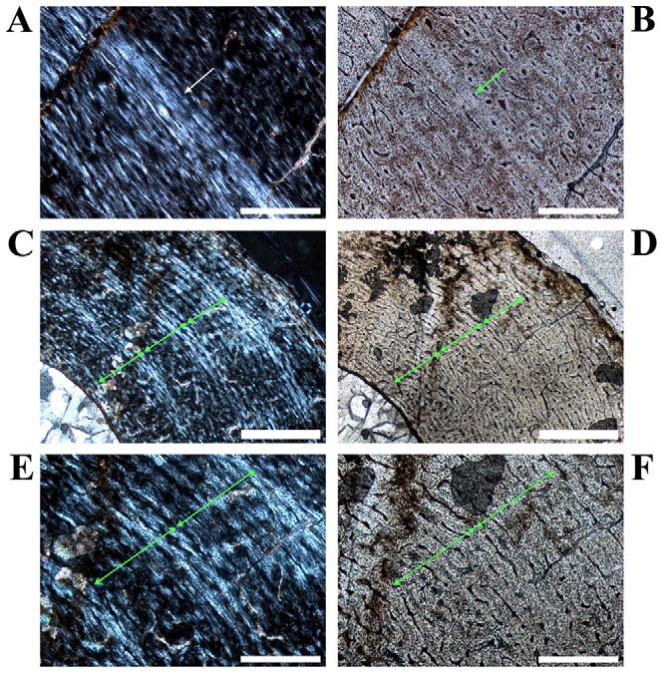
Growth cycles in *Dysalotosaurus* tibiae. A–B: Large tibia SMNS T3, A – Close up of the anterior bone wall with a slow growing zone flushing externally with an annulus (arrow) and a LAG (at the internal edge of the former), under polarized light. B – The same as in A under normal light. The arrow marks again the annulus. The LAG is visible as up to two thin lines at its internal edge. C–F: Smaller tibia GPIT/RE/3724, C – Anteromedial corner under polarized light with up to five slow growing zones. The three middle growth cycles, consisting of a fast and a following slow growing zone, are completely visible (marked by three double-headed arrows). Whether the utmost slow growing zone is complete or not cannot be verified. D – The same as in C under normal light. Fast and slow growing zones are again difficult to distinguish. Apart from using polarized light, only minor differences in the organization of primary osteons are visible. E – Magnification of C with the two external arrows included. F – Magnification of D with the two external arrows included. Scale bars = 1 mm in C–D. Scale bars = 500 µm in A–B, E–F.

The zonation pattern is also similar to that of the femora, with very few annuli/LAGs and with growth cycles mainly consisting of fast and slow growing zones (Figs. 1F, H; 9A, E; 10; Tab. 2). The growth cycles are best preserved in the anterior and/or medial walls. A transition from distinct growth cycles internally to a uniform slow growing area externally, as occurs in five large femora, is not visible in the two large tibiae.

Secondary remodeling is even rarer than in femora. The only area with preserved secondary osteons is the anterolateral corner of large (SMNS T3; GZG.V 6791, see Tab. 2) and more distal sections. Scattered examples are found mainly in the outer area of the CCCB wedge and within the Anterolateral Plug (Fig. 9H).

**Figure 11:**
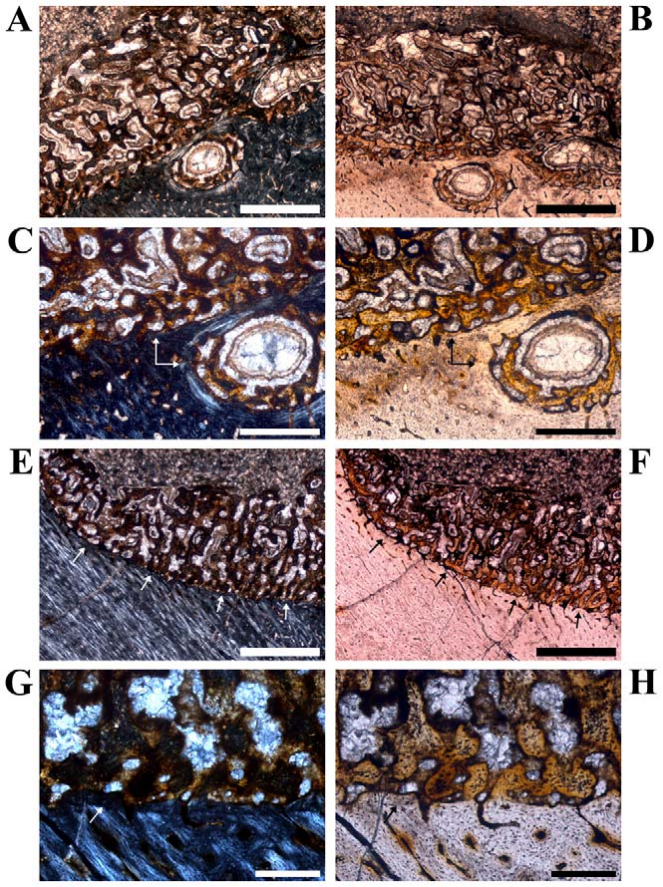
Details of medullary bone found in a single tibia of *Dysalotosaurus*. A–H: Large tibia SMNS T3 with images of the preserved medullary bone tissue at the anterior edge of the marrow cavity. See also Fig. 1G–H for an overview, A – The strongly cancellous medullary bone tissue (mainly in brown colors, under polarized light) is also developed within two large cavities at the edge of the marrow cavity. The difference to the primary bone tissue at the bottom and the CCCB at the lower right is striking. B – Approximately the same as in A under normal light, only slightly rotated image. The strong difference of the medullary bone tissue to the tissue types within the bone wall is still well visible. C – Close up of one part visible in A and B under polarized light. The separation between medullary bone and the actual bone wall tissues is marked by an endosteal layer (arrows). D – The same as in C under normal light. E – Overview of the medial part of the preserved medullary bone tissue under polarized light. The endosteal layer is marked by arrows. F – The same as in E under normal light. G – Magnification of E between its two central arrows under polarized light. The endosteal layer is again marked by an arrow. Note the resorptive nature of this part of the marrow cavity before the development of the endosteal layer. H – Same as in G under normal light. Scale bars = 1 mm in A–B, E–F. Scale bars = 500 µm in C–D. Scale bars = 200 µm in G–H.

In one of the large tibial cross sections (SMNS T3), at the anterior edge of the marrow cavity, an unusual bone tissue is preserved (Figs. 1G–H; 11). It is strongly cancellous with irregularly shaped caverns of various sizes. It is weakly birefringent under polarized light. It is also clearly separated from the compact bone wall by an endosteal layer (Fig. 11C–D, G–H). Some of this tissue was also found inside two large caverns within the CCCB-wedge (Fig. 11A–D). All these features, and the absence of any external pathologies (including a thickening of the bone wall or bilaterally symmetrical occurrence of unusual tissue as a sign for osteopetrosis [60], [61]), indicate that this tissue belongs to the endosteal type of tissue called medullary bone, which has already been documented in three other dinosaur taxa [62], [63] (but see [60]). This tissue is known among living vertebrates only in birds and it functions as storage for the calcium needed for the development of eggs in breeding females. Thus, medullary bone tissue is also a marker for sexually mature females around the breeding period [63].

### Ontogenetic Stages in the Tibiae

The recognition of distinct ontogenetic stages in the tibiae is more difficult than in the femora, because there are fewer tibial sections available, and because most of the available specimens, belonging to a medium size range (see stage 3 below, Tab. 2), are probably of the same immature stage. However, the differences between these and the younger and older stages are substantial, owing mainly to the preserved number of growth cycles and the number and distribution of secondary osteons.

#### Stage 1 or Embryonic Stage

As in the femora, this stage is unknown in the tibiae.

#### *Stage 2 or Early Juvenile Stage*

**Figure 12:**
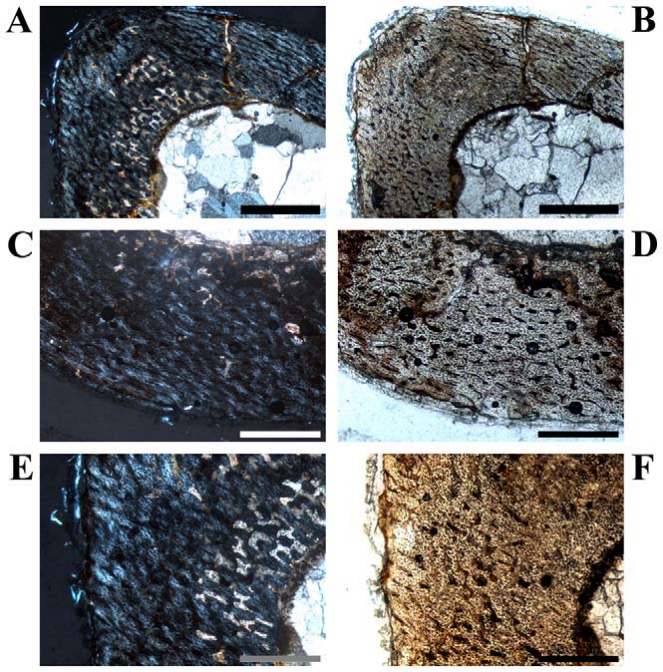
Bone histology of the smallest preserved tibia of *Dysalotosaurus*. A–F: Early juvenile tibia GPIT/RE/3795, A – Overview of the anterolateral corner under polarized light. CCCB and the Anterolateral Plug are absent. The interior part of that corner is altered by preservation (see also Fig. S1). B – The same as in A under normal light. C – The posterior wall is well vascularized and the primary osteons are plexiform to reticular in arrangement. The degree of organization as well as of the birefringence seems to increase towards the external surface, under polarized light. D – The same as in C under normal light. E – Magnification of A under polarized light. F – Magnification of B under normal light showing many simple vascular canals oriented radially. Scale bars = 1 mm in A–B. Scale bars = 500 µm in C–F.

Probably only a single tibia belongs to this stage (GPIT/RE/3795; Fig. 12; Tab. 2). The different units of the cross section differ only slightly from each other. An Anterolateral Plug, secondary osteons, CCCB, an endosteal layer, and resorption activity of the marrow cavity are all absent. Primary osteons are present, but they are still under development. Simple, laminarly organized, longitudinal canals are common, but radial orientations are also visible in the anterolateral corner (Fig. 12A–B, E–F). If at all present, only the beginning of the first slow growing zone is visible at the outer edge of the bone wall.

#### Stage 3 or Late Juvenile to Sexually Immature Stage

These cross sections possess much better differentiated units including the Anterolateral Plug, which occur in distal sections within the shaft. CCCB, secondary remodeling, and resorption by the marrow cavity are observed in some sections. The knitted pattern is now only preserved in the inner cortex, whereas primary osteons are now well developed and widely distributed. At least two to three growth cycles are present (Fig. 1E–F; 10C–F).

#### Stage 4 or Sexually Mature Stage

The two largest samples (SMNS T3; GZG.V 6791) belong to this stage. The cross-sectional units are strongly differentiated and the bone wall thickness is highly variable (Fig. 1G–H). The CCCB tissue forms a large wedge, which reaches far into the cortex anterolaterally. There is a distinct swirl-like Anterolateral Plug within the anterolateral corner (Fig. 9E). Simple juvenile vascularization is preserved only as a relict in some of the innermost parts (Fig. 9A–B). Secondary osteons are more abundant within the Anterolateral Plug (Fig. 9E, G). Primary osteons are dense and numerous. The number of growth cycles exceeds three. Finally, medullary bone may be found in one of the cross sections of this stage (Fig. 11).

### Bone Histology of the Humerus of *Dysalotosaurus*

#### Description

**Figure 13:**
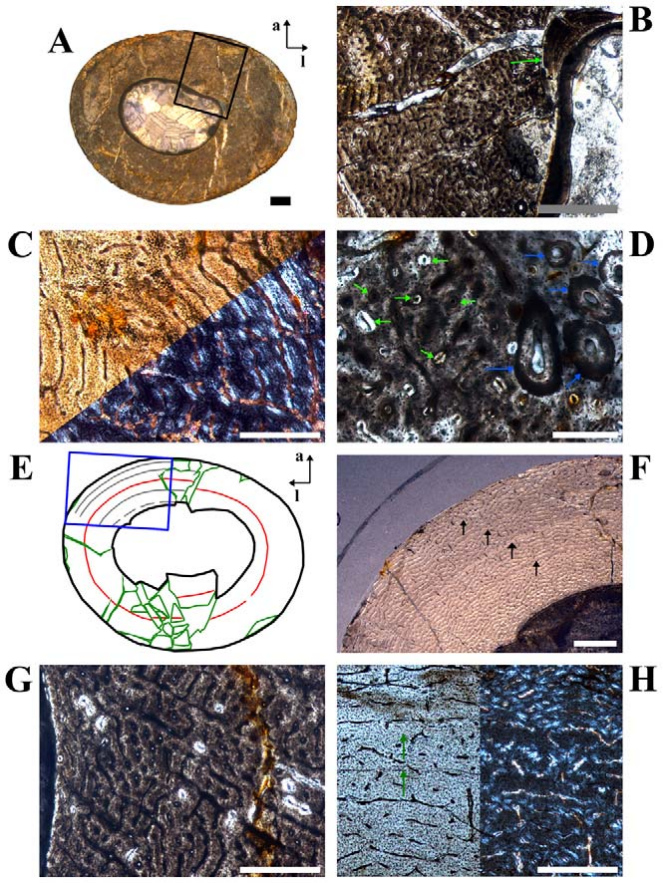
Bone histology in *Dysalotosaurus* humeri. **A–B: GPIT/RE/4402, A – Orientated overview (a = anterior, l = lateral) under normal light.** Note the differences in bone wall thickness and between the shapes of the marrow cavity and the whole cross section of this proximally cut section. B – Magnification of the framed area in A (rotated anti-clockwise by app. 120°) under normal light. The thick wedge of lamellar bone of the endosteal layer is marked by an arrow. Note the large amount of small longitudinal primary osteons. C: GZG.V 6664, Close up of the anterolateral corner with circumferential primary osteons separated by very thick cords of matrix. These relations in thickness together with areas of very high concentrations of osteocyte lacunae (left centre of image) are restricted to this cross sectional unit, under both polarized and normal light. D – Magnification of lower centre of B under normal light. The marrow cavity is close by in the direction to the right. Small primary osteons with only a single ring of lamellar infilling are marked by green arrows. Secondary osteons are marked by blue arrows. E–F: GZG.V 6569, E – sketch with the external edges of slow growing zones marked in gray and a LAG marked in red. The dashed line represents an unsecured slow growing zone. F – Image of the framed area in E under normal light. The four secured cycles of E are marked here by arrows. Note the increasing organization of primary osteons towards the periphery and the slight differences between the fast growing zones and the often rather thin slow growing zones. The LAG is marked by the wide-headed arrow. G: GPIT/RE/4402, the internal area of the posterior bone wall is rotated 90° clockwise relative to A and shows many inclined radial canals. These canals are perpendicular to the surface medially. H: GPIT/RE/4877/8929, Arrows indicate two closely located LAGs, under both polarized and normal light. Scale bars = 1 mm in A–B, F. Scale bars = 500 µm in C, G–H. Scale bars = 200 µm in D.

The shape of the cross sections varies from a lateromedially wide and flat oval outline distally to an almost circular oval shape more proximally (Fig. 1I–L). CCCB is very rare and only visible in various units in the most distal sections and in the anterolateral part in the most proximal sections. More common is the development of an endosteal layer, although it never surrounds the marrow cavity completely. Proximal sections often possess a thick but short wedge of endosteal bone in the anterolateral corner of the cavity (Fig. 13A–B).

The bone matrix of the primary compact bone wall consists mainly of fibrolamellar bone tissue, although the anterolateral corner can be built by parallel-fibered tissue in some of the more proximal sections (Fig. 13C). However, this Anterolateral Plug is only visible in mid diaphyseal and proximal sections and is much less distinct than in femora and tibiae.

Primary osteons are numerous and dense, but there are high numbers of relatively smaller and longitudinal osteons with a strongly birefringent single ring of lamellar infilling (Fig. 13D). Such small primary osteons are absent in femora and tibiae, but the relative amount of well-developed larger primary osteons as well as their density is the same. The dominant type is again the laminar organization (Fig. 13F, H). In some proximal sections, convoluting radial canals can be found, which often extend throughout the whole thickness of the cortex (Fig. 13G).

Annuli/LAGs are more abundant than in femora and tibiae, but their distribution is still very inconsistent (Fig. 13E–F, H).

Secondary osteons are very rare. They are mainly located at the edge of the CCCB in the most distal or proximal sections, but they mainly occur close to the internal margin of the anterolateral corner along the edge of the short endosteal layer (Fig. 13D) or within the Anterolateral Plug.

### Ontogenetic Stages in Humeri

The differentiation of humeral cross sections into ontogenetic stages is much more ambiguous than in the femora and tibiae. The only clear features are the size and the number of growth cycles.

#### *Stage 1 or Embryonic Stage*

As in the other sectioned elements, this stage is not preserved.

#### *Stage 2 or Juvenile Stage*

The smallest sections with not more than a single growth cycle belong to this stage (Tab. 3; Fig. 13A–B, D, G). The slow growing part (zone, annulus, or LAG) exists close to or at the outer rim of the bone wall. The degree of organization of the vascular canals is low, so that plexiform to sometimes reticular tissue type predominates.

**Table 3:** Basic dataset of all cross sections of humeri used for correlations.

Labels	Cutting level	MWDC	Ant-post Ø	Med-lat Ø	Ant-post cavity	Med-lat cavity	BWT max	BWT min	Number growth cycles	Number LAGs/annuli	Ontogenetic stage	Age in years
SMNS H 2	proximal to diaphysis	11.3	6.2	8	3.2	4.8	1.9 anterolateral	1.1 anteromedial	―	―	juvenile	1
GPIT/RE/4526	proximal to diaphysis	13.2	―	8.8	4.9	5	―	―	1	1	juvenile	2.6
GPIT/RE/4402	proximal to diaphysis	16.3	8.8	11.1	3.4	4.8	3.8 lateral	2.3 anteromedial	1	1	juvenile	5.4
GPIT/RE/4262	app. diaphysis	23.3	―	16.2	6.7	7.7	―	―	1	1	sex. mature	10.8
GZG.V6569	distal to diaphysis	24.2	12.7	14.8	―	8.8	―	―	5	1	sex. mature	11.8
GZG.V6664	proximal to diaphysis	24.8	15.2	16.3	8.1	9.1	4.2 lateral	―	4	1	sex. mature	12.7
GZG.V6223	distal to diaphysis	26	15.3	19.6	8.2	12.5	4.2 anterior & posterior	3.1 lateral & medial	4	2	sex. mature	15.3
GPIT/RE/4877/8929	proximal to diaphysis	30.4	16.1	20.8	7.3	11.2	5.3 anterolateral	3.2 anteromedial	4–5	3	sex. mature	19.3
GPIT/RE/6416	distal to diaphysis	31.7	17.2	23	10	14.4	4.6 posterolateral	2.9 anterior & lateral	3–4	2	sex. mature	>20

Explanation of heading-abbreviations: MWDC –Mediolateral width at the deltopectoral crest; the remaining headings as in Table 1. The age in years was estimated by the comparison of relative positions within the size-frequency distributions of femora and humeri, respectively. All data in mm.

#### Stage 3 or Post-Juvenile Stage

All remaining cross sections belong to this stage and a further subdivision is not possible. The number of growth cycles exceeds one and the laminar vascular pattern predominates (Tab. 3; Fig. 13C, E–F, H).

### Bone Histology of the Fibula of *Dysalotosaurus*

Due to the scarcity of preservation of fibulae, cross sections could only be produced from levels very close to or within their proximal metaphysis. Therefore, periosteal compact bone is, if at all, often present as a thin layer surrounding parts of the bone wall externally and it was impossible to get a truthful count of growth cycles.

**Figure 14:**
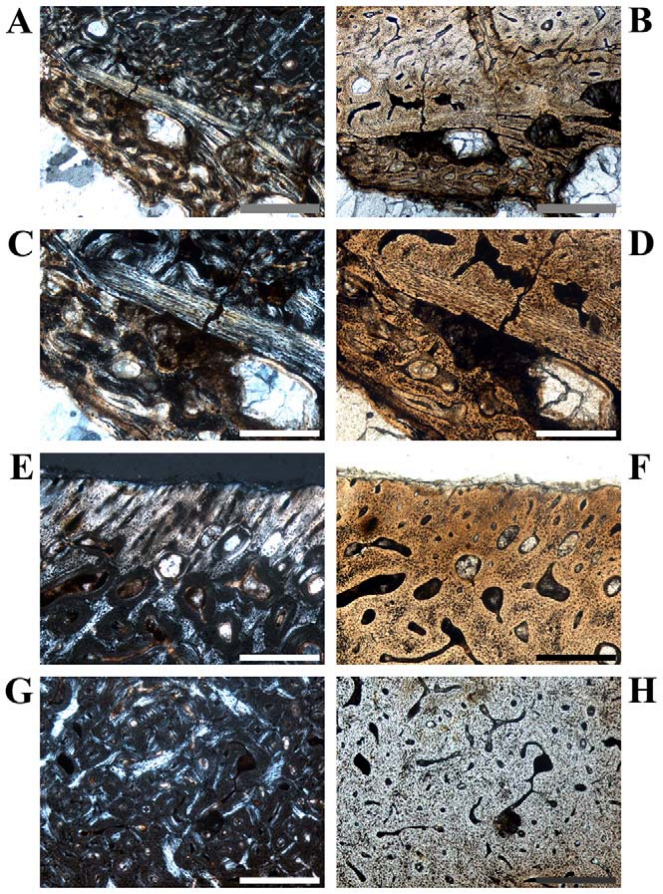
Variation in the bone histology of a single large fibula of *Dysalotosaurus*. A–H: Large fibula GPIT/RE/5109, A – Internal area of the posteromedial bone wall, under polarized light, with a thick endosteal layer separating the possible medullary bone tissue from the bone wall. B – Same as in A under normal light and slightly rotated. C – Magnification of the left centre of A under polarized light. The CCCB immediately external to the endosteal layer is much stronger birefringent than the medullary bone tissue. D – Same as in C under normal light. E – External part of the medial wall with only small simple vascular canals and some weakly developed primary osteons. The secondary osteons are rather large and different stages of development are present. F – The same as in E under normal light. G – Close up of the posterior corner with numerous secondary osteons obscuring most of the remaining CCCB, under polarized light. H – The same as in G under normal light. Scale bars = 1 mm in A–B. Scale bars = 500 µm in C–H.

The overall shape of the cross sections is oval to kidney-like with very thick and strongly curved bone walls anteriorly and posteriorly. Most of the outer rim of the marrow cavity is poorly defined because of wide cavernous spaces surrounded by a loose network of trabeculae. An endosteal layer can only be observed along the thinner lateral and medial walls. This band of lamellar bone is very thick posteromedially (Fig. 14 A–D).

The thin layer of periosteal primary compact bone consists of fibrolamellar bone tissue, although the primary osteons are often relatively small and scattered. Endochondral bone tissue is often developed between this peripheral fibrolamellar bone and the internal CCCB.

The medial wall differs strongly from the other units, because it is heavily altered by dense Sharpey's fibers, so that the area is strongly birefringent under polarized light (Fig. 14E–F). The bone matrix seems to be completely metaplastic in origin and the vascular canals are simple, elongated, and oriented parallel to the Sharpey's fibers.

Secondary osteons are very common in these metaphyseal cross sections. The CCCB is not involved, but its external border and most of the endochondral tissue is strongly remodeled. Internal and mid cortical areas of the posterior corner may even consist of dense haversian tissue of at least two generations of secondary osteons (Fig. 14 G–H). The medial wall is affected by very coarse remodeling (Fig. 14E–F), because the scattered secondary osteons are much larger.

In the cross section of the large fibula GPIT/RE/5109, possible medullary bone is preserved internal to a part of the endosteal layer that fans out (Fig. 14A–D). The medullary bone tissue also differs from the thick layer of CCCB external to the endosteal layer by the lack of birefringent lamellar bone typical for the latter, by the complete lack of osteonal development, and by a much higher density of osteocyte lacunae within its reticular network.

### Bone Histology of the Prepubic Process of the Pubis of *Dysalotosaurus*

**Figure 15:**
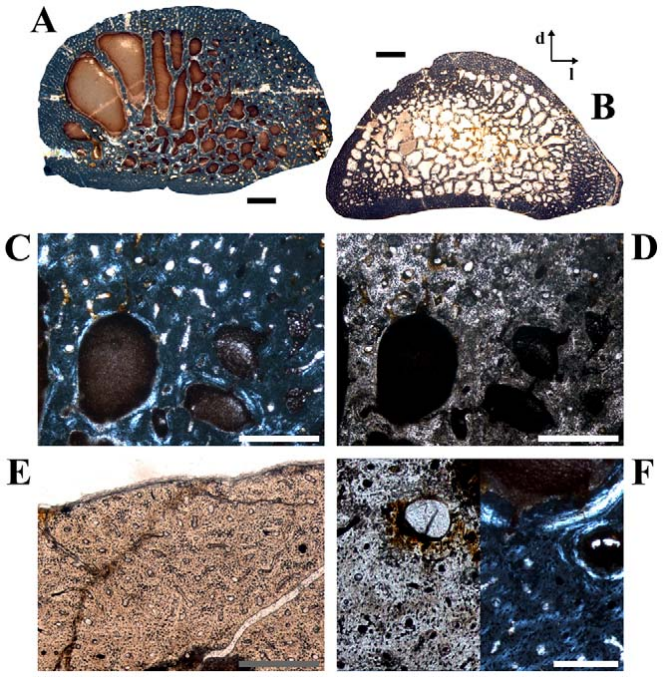
Orientated images of cross sections of prepubic processes (d = dorsal, l = lateral) of *Dysalotosaurus*. A: SMNS P17, proximal section under polarized light with decreasing size of the pseudocavities towards the lateral side. B: SMNS P19, distal section under polarized light. Note the double-layered order of the erosion cavities dorsally and similar single layers of cavities medially and ventrally, respectively. C–D: SMNS P17, C – Magnification of the dorsal centre of A under polarized light showing primary bone tissue with mainly longitudinal, small primary osteons even between the erosion cavities. The latter already possess layers of lamellar bone. D – The same as in C under normal light. E: SMNS P19, Close up of the dorsal primary bone wall with mainly longitudinal but well developed primary osteons. F: SMNS P17, Magnification of the lower centre of A with simple vascular canals and very small, weakly developed primary osteons, under both polarized and normal light. Resorption and secondary infilling of cavities is visible at the top of the image. Scale bars = 1 mm in A–B. Scale bars = 500 µm in C–E. Scale bars = 200 µm in F.

The sections cut directly at the maximum lateromedial width of the prepubic process have a wide oval shape (Fig. 15A). Sections taken more distally/anteriorly to the maximum width of the prepubic process have a triangular to lamp shade-like external outline (Fig. 15B).

The periosteal compact bone wall is very thin compared to the overall diameter of the cross sections. There is no consistent internal margin, because a single large marrow cavity is absent. However, some of the internal cavities are quite large. These cavities are always of resorptive origin, because remnants of periosteal compact bone are often still preserved in some of the thicker trabeculae (Fig. 15C–D).

This tissue consists of vascular fibrolamellar bone. Well-developed primary osteons are mainly visible in the dorsal and medial parts of the bone wall, but they are not very dense and mostly longitudinal in arrangement (Fig. 15E). Mainly ventrally, primary osteons are rare, relatively small, and weakly developed. Here, the matrix is often almost opaque and the often simple vascular canals are also longitudinally organized (Fig. 15F). Growth cycles are very rare, but there are at least one to two annuli/LAGs preserved in some sections.

Numerous small secondary osteons occur in the trabeculae between the large pseudo-cavities as well as in the internal areas of the periosteal compact bone wall, where they sometimes form haversian tissue. Their abundance decreases towards the medial side.

### Quantitative Results

**Figure 16:**
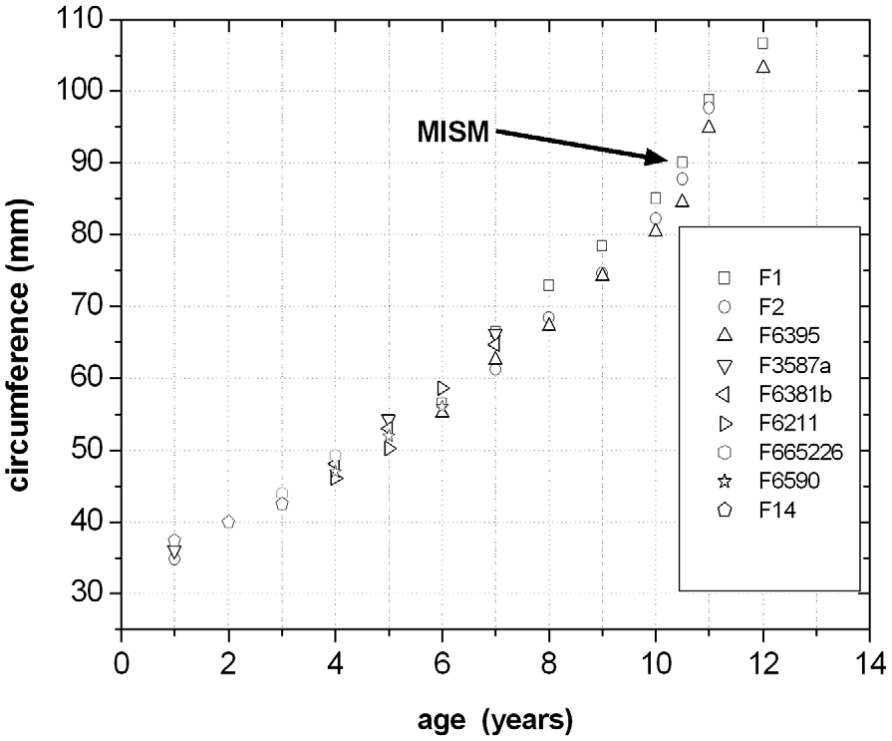
Fractional growth cycle values of femur group two are correlated to age. MISM = Mark of Initial Sexual Maturity. ‘F’ is the abbreviation for ‘femur’. Each of the following numbers corresponds to the respective specimens in Tab. 1. Some specimens were sampled at least twice so that additional letters (a, b) advert to the respective section used for this correlation.

**Figure 17:**
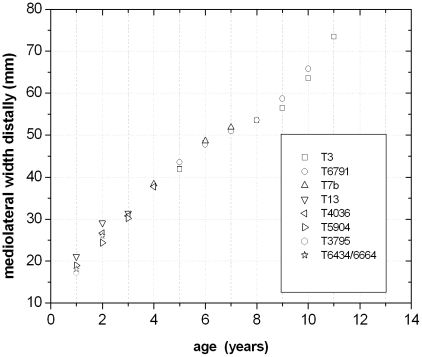
Fractional growth cycle values of tibia group one are correlated to age. ‘T’ is the abbreviation for ‘tibia’. Each of the following numbers corresponds to the respective specimens in Tab. 2. Some specimens were sampled at least twice so that additional letters (b) advert to the respective section used for this correlation.

**Figure 18:**
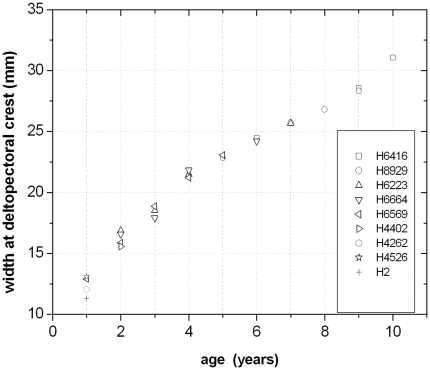
Fractional growth cycle values of the single group of humeri are correlated to age. ‘H’ is the abbreviation for ‘humerus’. Each of the following numbers corresponds to the respective specimens in Tab. 3.

**Figure 19:**
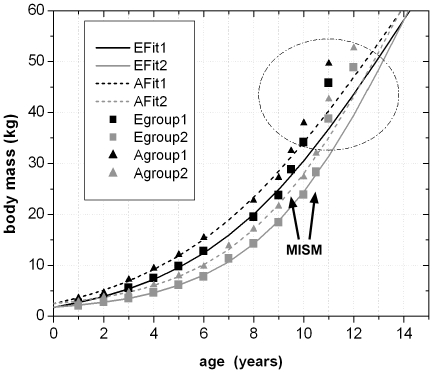
The nine correlated growth cycle values of femur group one and two were combined with the values of the MISM and were used for the calculation of four growth curves. All encircled values represent unsecured growth cycles external to the MISM and were plot into the diagram afterwards. The shift of these points onto their respective growth curves resulted in a graphical change of only one additional year in age in average. Thus, 13 years are finally represented by all visible growth cycle values. Abbr.: EFit1 – Growth curve of femur group one, calculated with body masses derived from Erickson & Tumanova [27]; EFit2 – Growth curve of femur group two, calculated with body masses derived from Erickson & Tumanova [27]; AFit1 – Growth curve of femur group one, calculated with body masses derived from Anderson et al. [64]; AFit2 – Growth curve of femur group two, calculated with body masses derived from Anderson et al. [64]; Egroup1 – Correlated fractional growth cycle values of femur group one, the respective body masses are derived from Erickson & Tumanova [27]; Egroup2 – Correlated fractional growth cycle values of femur group two, the respective body masses are derived from Erickson & Tumanova [27]; Agroup1 – Correlated fractional growth cycle values of femur group one, the respective body masses are derived from Anderson et al. [64]; Agroup2 – Correlated fractional growth cycle values of femur group two, the respective body masses are derived from Anderson et al. [64].

The combination and correlation of the fractional values of the growth cycles for each group of cross sections resulted in a quite consistent number of years represented by these cycles. Thus, the combined growth cycles in femur group one (sections from the top of the proximodistal shelf close to the middle of the shaft) represent 11 years, those of femur group two (sections from the base of the fourth trochanter) represent 12 years (Fig. 16), and those of tibia group one (sections well within the distal third of the shaft) represent 11 years (Fig. 17). The only group for humeri represents ten years recorded by all combined growth cycles (Fig. 18), although several cycles were probably not recognized (compare with Tab. 3). The remaining groups three and four in femora as well as group two in tibiae contain only three to four cross sections without enough preserved growth cycles for a secure correlation. The Mark of Initial Sexual Maturity (MISM) in femora always correlates with an age of approximately 9.5 years in femur group one and 10.5 years in femur group two (Figs. 16; 19).

To calculate the respective body masses for the correlated growth cycles with the Developmental Mass Extrapolation method [27], and to calculate the sigmoidal growth curves, it was necessary to calculate the maximum body mass. The largest femoral specimen (MB.R.2144) represents a body mass of 115.3 kg using the method of Anderson et al. [64] for bipeds. In the same way, the respective body mass at the MISM was calculated as 32.44 kg on average for femur group one and 31.96 kg for femur group two.

By using the first nine (femur group one) to ten (femur group two) secured growth cycle values, the respective values of the MISM, and the maximum body mass, four sigmoidal growth curves were created. The remaining growth cycle values, representing unsecured growth cycles external to the MISM, were plotted into the curves subsequently (Fig. 19). The manual shift of these values by one year on average resulted in the ideal fit to their respective growth curves. At the end, a total of 13 years of life of *Dysalotosaurus* are represented by the observed and correlated growth cycles in the femoral cross sections of groups one and two (Fig. 19).

**Figure 20:**
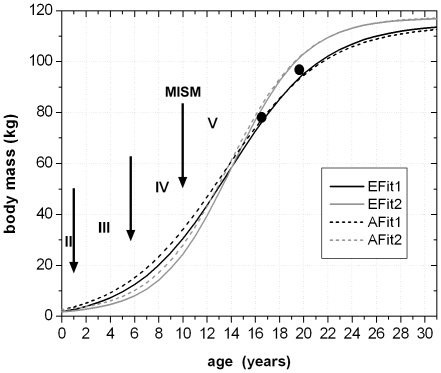
The four complete growth curves derived from the values shown in **Fig. 19**. Abbreviations for the curves are as in Fig. 19. The arrows separate the ontogenetic stages observed in the femoral cross sections: II – Early juvenile stage; III – Late juvenile stage; IV – sexually immature stage; V – sexually mature stage. The black point at app. 16.5 years of age represents the largest sampled femur. The black point at app. 19.5 years of age represents the third largest preserved femur.

The now known values of the four parameters of each of the four growth curves were used to calculate the respective values for all known femora of *Dysalotosaurus*. The largest sampled femur (SMNS F2, group two) would therefore represent an age of 16.5 years (body mass after [64]) or 16.3 years (body mass after [27]). The age of the third largest femur found in the collections (R12277) would then represent an age of 19.7 years (after [64]) or 19.3 years (after [27]) (Fig. 20).

**Figure 21:**
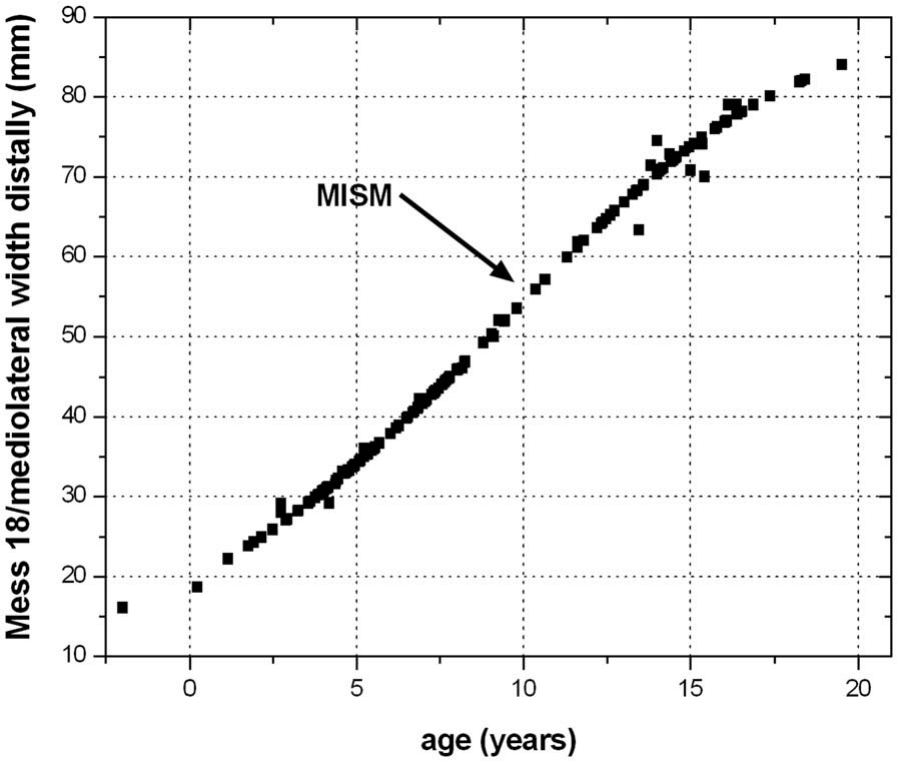
In contrast to the diagrams with body mass versus age, the MISM is almost exactly positioned at the inflection point in a curve with body size versus age. Measured and calculated values of the distal mediolateral width of femora are combined. The age values are an average of the respective values calculated by the methods of Anderson et al. [64] and Erickson & Tumanova [27].

The MISM is located well between the lower and middle third of the growth curves, if body mass is plotted versus age (Fig. 21). Thus, the growth rate of body mass is still accelerating after this mark and reaches its maximum in the 14^th^ year with a daily increase of 24 to 26 grams (for femur group two). However, by plotting the respective values of the distal mediolateral width of femora or their midshaft circumference (representing body size) versus age, the MISM is then located very close to the inflection point of the curve (between accelerating and decelerating growth rate: Fig. 21). Finally, the relative body size of *Dysalotosaurus* at the MISM reaches 62.1% for the femoral distal mediolateral width and 63.4% for the midshaft circumference when compared to the known maximum body size.

## Discussion

### Variation within Bone Tissues in *Dysalotosaurus*

Variation within bone tissues in *Dysalotosaurus* is exhibited between different individuals, within the ontogenetic series, within a skeleton, within a bone, and even within a cross section. This variation also clearly demonstrates that comparative bone histology is only significant when the sampling is standardized among several skeletal elements and the relative ontogenetic stage is considered (e.g. [6], [36], [38]).

#### Variation between Different Skeletal Elements

The bone wall of the main weight bearing long bones (femora, tibiae) of *Dysalotosaurus* are naturally thicker than in the sampled humeri, fibulae, and prepubic processes. Interestingly, the relative growth rate is also higher in these long bones compared to the other sampled elements, which is inferred from the overall development, density, and organizational degree of vascular canals (see e.g. [8], [36], [39], [41], [46]). Femora and tibiae possess a comparatively higher amount of well developed primary osteons and larger areas with plexiform or even reticular vascularization than humeri and prepubic processes. Thus, as in *Maiasaura* [36] and *Plateosaurus* [32], [33], different skeletal elements grow at different rates during ontogeny.

A possible explanation for growth rate changes may be the absolute size of the respective element within the skeleton combined with the degree of utilization, which includes two components: (1) the degree the element participates in weight bearing and (2) the functional demand on the bone. In the case of the biped *Dysalotosaurus*, the femur and tibia are the largest and primary weight-bearing bones intensively used for locomotion. The humerus is comparatively much smaller (in the only preserved individual ‘dy I’, exhibited in Berlin, app. 57% the length of its femur) and was likely not used in weight bearing or locomotion. It is therefore not surprising to find it less densely vascularized within a relatively thinner bone wall. The sampled prepubic process is even more different than the femur and tibia in these characters, because it serves only as muscle attachment site and is not involved in active movements or in bearing weight.

Similar tendencies are visible in other tetrapods, but it strongly depends on their respective skeletal bauplan. The humerus of the therapsid *Diictodon* reached higher relative growth rates than its femur [65], because it was probably used for digging in addition to weight bearing. This is also observed more extensively in the common mole (*Talpa europea*) by Enlow & Brown [66], where the large humerus is well vascularized and the much thinner cortex of the smaller tibia is almost avascular indicating the tibia had a much slower relative growth. It is not as simple in birds and pterosaurs, because the demand on active forelimbs, mainly for flying, against weight bearing hindlimbs is highly speculative. However, there are at least indications that the absolute size of bones in pterosaurs [67], in penguins [47], and in some dinosaurs (see e.g. [32], [36]) is correlated with relative growth rate in these groups as well. Although there are no subsumable differences in the vascularization pattern between elements in recent ratite skeletons, the flightless habit almost predicts much lower growth rates for the forelimb elements compared to the elements of the hindlimb [41]. This is also comparable to biped dinosaurs, such as *Allosaurus* (see e.g. [68]) and *Dysalotosaurus*, or facultative quadruped dinosaurs with a strong size difference between fore- and hindlimbs, such as *Scutellosaurus* [43].

Within a single limb, the bones of the stylopodium (humerus, femur) have higher relative growth rates than the bones of the zeugo- and autopodium, because the latter are often smaller in overall size and share functions, such as weight bearing or muscle activity, among each other. The absolute forces acting on each of them are therefore smaller than in the stylopodium. This is the case for the less vascularized radii and ulnae compared to the humeri and femora in *Thrinaxodon* [69] and to the femora in *Scylacops* [70], and for the ulnae of *Allosaurus* and *Tenontosaurus* compared to the other sampled bones of the respective studies [34], [68]. Nevertheless, whenever bones of the zeugo- and autopodium are fused (e.g. to the tibiotarsus and tarsometatarsus in birds), are much more prominent than their neighbors (e.g. the tibiae in many dinosaurs), or are exclusively used for powerful movements (e.g. the wing phalanges of pterosaurs), their relative growth rates should be more comparable to the bones of the stylopodium (see [41], *Dysalotosaurus* [68], respectively). In all these cases, the fused bones are also larger than usual.

In the end, the relative size of a bone in a skeleton reveals its importance in weight bearing and/or movement and its relative growth rate compared to other elements is therefore predictable to a certain degree.

#### Variation between Different Cross Sectional Units

Cross sections with very consistent outlines (especially distal and mid diaphyseal humeri; Fig. 1) reveal much less variation of bone tissues than cross sections with irregular outlines and acute corners, such as femoral sections (Figs. 1–6), distal tibial sections (Figs. 1G–H; 9; 12), and prepubic sections (Fig. 15). Some of the intrasectional variation is caused by differences in bone wall thickness. The thicker posteromedial and posterolateral corners in femora and the anteromedial corner and medial bend in tibiae have a high density of weakly-organized primary osteons (e.g. Figs. 3A–B; 9C) and osteocyte lacunae. The collagen fibrils in these areas are also hardly organized so that there is only a weak birefringence under polarized light. Finally, the slow growing zones are weaker and the distances between them are larger than in the thinner bone wall units (see below; Fig. 1H, L). The opposite trend of the noted features takes place in the latter (in the anterior corner of femora and in the anterolateral corner of tibiae) (Figs. 2E–F; 3E–F; 8C; 9A–B, E). A similar pattern can be seen in the largest sampled femur of *Dryosaurus altus* [52].

The variation in relative growth rates due to variable bone wall thickness is superimposed by another source of variation in femora, distal tibiae, and proximal humeri. The anterior corner in distal femora, the anterolateral corner in distal tibiae, and sometimes the anterolateral corner in proximal humeri, consist of an internal wedge of CCCB (femora, tibiae) or of endosteal lamellae (mainly humeri). The external periosteal regions possess here well organized primary osteons in a low density, osteocyte lacunae are also rarer than in other units, and the collagen fibrils are mainly transversely organized (Fig. 2E–F; 3E–F; 13C). All growth cycles (including annuli/LAGs) are closer together (Fig. 1H, L). The bone wall of the opposite side of the cross sections (posterior bend in femora, medial sides in distal tibiae and proximal humeri) is distinctly resorbed internally by the marrow cavity (Figs. 1B, D, H, L; 2A–B; 5E) and is more similar to thick bone wall units (Figs. 1B, D, H, L, 2A–D; 3A–B; 5C–D, F–G; 9C; 10C–F; 13G). Thus, the latter units were deposited by much higher relative growth rates than the former units.

These differences in growth rate of opposing cross sectional units are explained by the drift of the marrow cavity towards the side with the suggested higher relative growth rate. The combination with the bending orientation of the respective long axes of the bone shafts indicates that the marrow cavity always drifts from the convex side of the long axis to the concave side to maintain the overall bone wall thickness during growth. The convex side of the long axis is located anteriorly in femora and laterally in distal tibiae and proximal humeri, respectively. This also explains why there is still unresorbed CCCB left in the mentioned units of relative slow growth, because this metaphyseal tissue is necessary for a consistent bone wall thickness during ontogeny [71]. For the same reason, juvenile bone tissue (small longitudinal primary osteons, knitting pattern of the matrix) is still preserved in the internal areas even in respective units of large cross sections (Figs. 3G–H; 6E–H; 8C; 9A–B, E–G). The typical intrasectional variation caused by osseous drift is well described in Enlow [71] for rats and monkeys and is also shown for *Varanus* (see figure 2E in [40]) and for the small lizard *Gallotia* (see figure 13 in [5]). In contrast, this typical variation is rarely described in fossil tetrapods, although it is documented in the multituberculate mammal *Nemegtbataar* (see figures 6 and 7 in [72] and indicated in the dinosaurs *Scutellosaurus* (see figure 2 in [43] and *Psittacosaurus* [27]). As a result, cortical drift is supposed to be the normal case in long bones with a bent long axis [8], [71] and should be considered before histological sampling, due to its strong influence on the microstructure and on estimating growth rates.

The described special bone tissue of the Posterolateral Plug in femora (Figs. 4A–D; 5C–E), of the anterolateral corner in tibiae and humeri (Figs. 9E, H; 13C), of the medial wall in fibulae (Fig. 14E–F), and of the lateroventral corner in prepubic processes (Fig. 15A–B), are suggested to be the result of muscle and/or tendon forces acting on these cross sectional units. This is indicated by the relationship of these special structures with external processes or attachment sites for muscles. The tissue structures also display the potential orientations of the acting muscle forces, because Sharpey's fibers are most abundant in these units and the vascular canals are often oriented in a dominant direction. These Plugs are also very restricted with sharp borders (Figs. 4A–B; 5E; 9E; 15B) and show more secondary remodeling. Scattered secondary osteons are sometimes even developed close to the external surface, which is very unusual for the ‘normal’ bone tissue in *Dysalotosaurus* independently of ontogenetic stage.

Such unusual restricted areas in cross sections are already mentioned for the femur in *Hypsilophodon* and described for the femur in *Iguanodon* [73]. There were also sharply delimited and more strongly remodeled areas (also visible in *Hypacrosaurus* [38]) in possible connection with muscle attachment sites. As in *Dysalotosaurus*, these special areas can also be sharply restricted to a certain level in the shaft and vanish over a short distance within the shafts long axis. Possible Plug-like structures are mostly known in the literature as local areas with unusually intensive secondary remodeling almost reaching the external surface (e.g. [30], [34], [36], [38], [52]). Horner et al. [36] already noted the possibility of muscle strain as a reason for these above-average remodeled areas, which was also pointed out by Currey [74].

#### Variation of Growth Cycles

The number, relative distances, and developmental degree of growth cycles are highly variable in *Dysalotosaurus*. Their number is naturally strongly influenced by ontogeny (the larger/older the more) and by the primary bone wall thickness. This can be seen between different elements of the skeleton. The thickest primary bone walls are developed in femora and tibiae with 12.5 and 11 mm, respectively. These elements preserve the highest number of growth cycles, which counts up to nine in the largest sections alone and up to 12 after ontogenetic correlations in all sections. Humeri, which have a maximum primary bone wall thickness of 5.3 mm in the samples, have only up to five cycles in a single section and up to ten after the correlation. The much thinner primary bone wall in the prepubic process can preserve only two cycles at maximum. The relative distances between growth cycles are also dependent on the cutting level within the shaft, because the average thickness of the periosteal bone wall is increasing towards the mid diaphysis and the portion of CCCB at the total bone wall thickness is here insignificant [8], [71]. The resulting differences in the course of calculated growth curves derived from these distances are even stronger between cutting levels than between methods for calculating body mass (Fig. 20).

In contrast to the results of Chinsamy [18], there are indeed annuli/LAGs preserved in *Dysalotosaurus*, but they are rather rare, especially in femora (Tab. 1). They are slightly more abundant in tibiae and prepubic processes and most abundant in humeri (Tabs. 2; 3). There is also no distinct pattern predicting the occurrence of annuli/LAGs, because a medium-sized femur can possess a LAG and a large femur none at all (Fig. 1B, D). In tibiae and humeri, the number of LAGs increases with increasing bone wall thickness, but this is the same pattern as for all growth cycles, and LAGs are only part of them (see e.g. Fig. 13E–F). Interestingly, some of the prepubic processes, with their extremely thin primary bone wall, possess more annuli/LAGs than the thick-walled femora.

Thus, the development of annuli/LAGs in the sampled skeletal elements of *Dysalotosaurus* seems to be dependent on several factors, where relative growth rate (the lower the more) might be dominating over bone wall thickness. In elements with relatively high growth rates (femora, tibiae), only unfavorable environmental conditions (e.g. long draughts) or dramatic events in the individual's life history (e.g. injury, disease) may have resulted in the rare development of annuli/LAGs.

The relatively random and rare formation of clearly defined annuli/LAGs is in striking contrast to the pattern seen in many other dinosaurs. In theropods (e.g. [25], [28], [75]), mainly primitive and/or smaller sauropodomorphs (e.g. [29], [32]), and some ornithischians studied (e.g. [27], [34], [36], [38]), annuli/LAGs occur much more regularly and not as an exception, as in *Dysalotosaurus*. Especially large and derived sauropods have much weaker cycles, such as polished lines [57] or zonal differences in vascularization [37], [56], [76], which are assumed to be annual markers as well.

**Figure 22:**
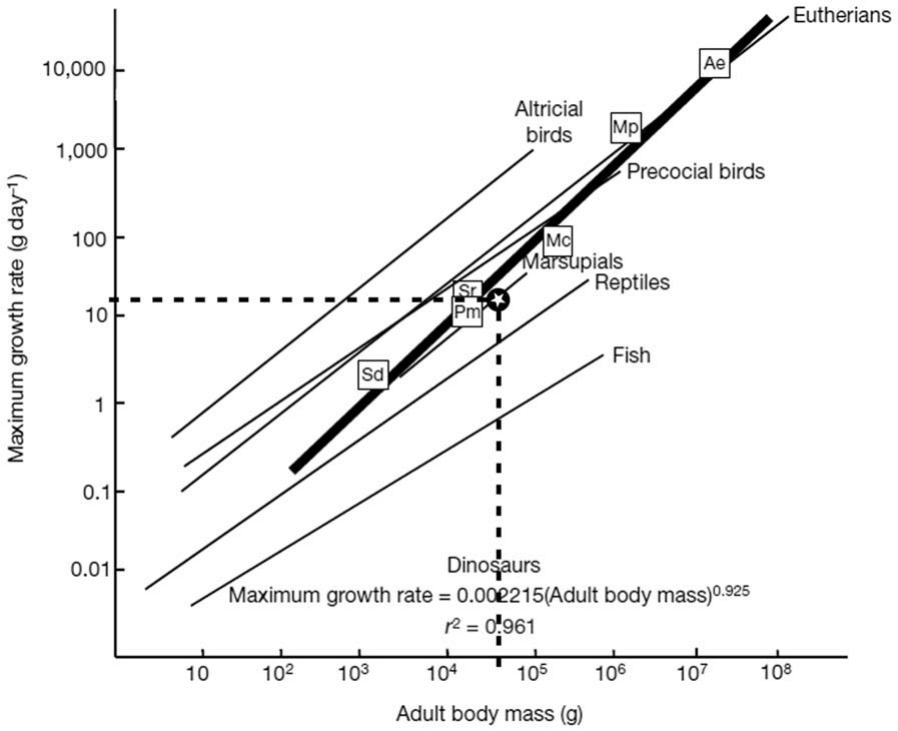
By comparing the maximum growth rate of *Dysalotosaurus* with other dinosaurs and recent animals, it is located close to the regression line for dinosaurs and is very similar to large marsupial mammals (modified from [**77**]). Abbr.: Sd – *Shuvuuia deserti*; Pm – *Psittacosaurus mongoliensis*; Sr – *Syntarsus rhodesiensis*; Mc – *Massospondylus carinatus*; Mp – *Maiasaura peeblesorum*; Ae – *Apatosaurus excelsus*.

None of these studies have mentioned the kind of growth cycles found here. Their identity as possible annual markers is now, however, unambiguously demonstrated. Despite the often relatively weak appearance (Figs. 2A–D; 5C–H; 10E–F; 13F), the cyclic occurrence of fast and slow growing zones is striking. As for annuli/LAGs: (1) their preserved number increases with related body size and is quite constant (with a maximum deviation of 2) throughout a single ontogenetic stage of a certain element (see also Tabs. 1–3); (2) the thickness of the slow growing zones is relatively constant, whereas the fast growing zones become thicker in the thick bone wall units and thinner in the thin bone wall units; (3) the zonation becomes weaker in thicker bone wall units and more distinct in thinner units of a cross section; and (4) the plot of the maximum growth rate with age, which is derived from the correlated growth cycles under the assumption of their annual signal, fits almost perfectly into the linear regression line of maximum growth rates developed for dinosaurs (Fig. 22, see also [7], [77], [78]). Cyclical fluctuations found in juvenile *Maiasaura* [36], in *Hypacrosaurus* [38], and in *Coelophysis* [79] are probably another kind of growth cycles, but their significance as annual markers is questioned by these authors and has still to be proved.

It is important to note that the type of growth cycles described for *Dysalotosaurus* probably exists in a wider range of taxa, because the cyclicity between zones of oriented collagen fibrils is also mentioned in *Alligator* ([59] see figures 2J–L; 3I–K; 4 therein), and is probably present in an extinct crurotarsian (pers. comm. Bronowicz, 2009) and in *Tenontosaurus* (pers. comm. Werning, 2010). Thus, this kind of growth cycles will probably be found in more tetrapods in the future and should provide age estimations especially in taxa with an otherwise poor record of annuli/LAGs.

### Correlation and Comparison of Ontogenetic Growth Stages

Since all the sampled elements are isolated and microstructural details vary between different elements of a skeleton, the correlation of ontogenetic stages in femora, tibiae, and humeri of *Dysalotosaurus* is only preliminary.

**Figure 23:**
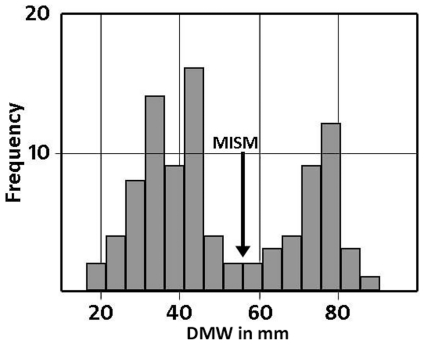
Size-frequency distribution of all measured right femora. The MISM is located at a femoral distal mediolateral width (DMW) of app. 55 mm (compare the DMW values with age in Tab. 1).

The second ontogenetic stage of all three elements (early juvenile or juvenile stage; Figs. 8A–D; 12) compares favorably, because each section belongs to the smallest available specimens and is located close to, or at, the left margin within the respective size-frequency distribution (Fig. 23). Furthermore, primary osteons are often incompletely developed (in humeri more advanced but very small; Fig. 13B, D), there is not more than one completed growth cycle, secondary osteons are extremely rare, and histological differences between sectional units are weak (Fig. 8A–D; 12). This correlated juvenile stage is similar to large nestlings in *Maiasaura* [36], to small juveniles in *Orodromeus* [52], and is located in between the perinate and juvenile stages of *Dryosaurus* [52].

The correlation of the older stages is more difficult, because there are different numbers of distinguishable stages in femora, tibiae, and humeri. Femora in the third and fourth stage of development (late juvenile (Figs. 3E–F; 6F; 8E–H) and sexually immature stages (Figs. 1C–D; 2E–F; 5A–B; 6G–H) are correlated with the third stage of tibiae (late juvenile to sexually immature stage (Figs. 1E–F; 10C–F), and the post-juvenile stage of humeri (Figs. 1I–L; 13A–B, D, G). Individual cross sections in humeri are only assignable to either sexually immature or sexually mature stages by their absolute size within the two-peaked size-frequency distribution of the *Dysalotosaurus* herd (Tab. 3). The respective cross sections of femora, tibiae, and humeri possess more than one growth cycle (up to five in the fourth femoral stage), the vascular pattern of vascular canals is mainly laminar to plexiform, primary osteons are abundant and well developed, secondary osteons, Plug structures, and osseous drift are present, and the cross sectional units are well diversified (less prominent in humeri). The closest similarities to described growth stages of other ornithopods were found in the large juvenile and subadult stages of *Orodromeus* [52], the juvenile and smallest subadult stages of *Dryosaurus* [52], and the juvenile stage in *Maiasaura* [36]. Both the late juvenile stage and sexually immature stage of *Dysalotosaurus* femora are also similar to the subadult stage in *Orodromeus* and to the small subadult stage in *Dryosaurus*.

The last represented ontogenetic stage is considered here as the sexually mature stage. This is clearly different to somatic maturity, because none of the sampled specimens show an External Fundamental System (EFS) as a sign for ceasing growth [6], [7]. In that sense, all sampled large specimens would represent somatically subadult individuals. The differentiation to younger stages is unambiguous in femora and tibiae, but only the absolute size and the position within the two-peaked size-frequency distribution of the *Dysalotosaurus* herd are helpful in humeri (Fig. 23; Tab. 3). Shared features of the sexually mature stage are well diversified cross sectional units with strong differences in bone wall thickness (less distinct in humeri), numerous growth cycles (up to nine in femora, seven in tibiae, five in humeri), often interrupted by strongly developed Plug structures (Figs. 1B, H; 4A–B; 5C–E; 9E), numerous and dense primary osteons, more abundant secondary osteons (Figs. 2G–H; 4C–D; 9H), and highly advanced osseous drift (Figs. 1B, H, L; 2A–B; 5E; depends especially in humeri on cutting level). This ontogenetic stage is comparable to the subadult stage in *Orodromeus* and the medium-sized subadult femur of *Dryosaurus* [52]. It does not match the subadult stage in *Maiasaura* due to the lack of extensive remodeling in the deep cortex and the lack of a starting EFS [51].

The ontogeny of the bone histology in *Dysalotosaurus* is most similar to *Dryosaurus* [52] regarding the overall size of skeletal elements as well as the respective cross sectional dimensions, vascularization pattern, and degree of secondary remodeling.

*Orodromeus*, on the other hand, reveals a vascularization pattern, which is usually found in skeletal elements of *Dysalotosaurus* with relatively lower growth rates, such as humeri or prepubic processes (Figs. 13; 15E–F). There, mainly longitudinal and smaller primary osteons are common, which are well described for *Orodromeus* [43], [52]. LAGs are also more common as in *Dysalotosaurus* and a possible EFS is known, which indicates nearly cessation of growth in the somatically mature adults. It confirms that this ornithopod, which has reached a smaller maximum body size than *Dysalotosaurus*, grew with a lower overall growth rate than the latter genus (other examples are e.g. [41], [43], [77], [80]).

The opposite case is the much larger hadrosaur *Maiasaura*. The vascularization pattern is not very different, but the thicker primary bone walls experienced more intensive secondary remodeling. Large and widespread resorption cavities or dense Haversian bone, which can obscure the primary bone in the deeper cortex, is completely unknown in the sampled elements of *Dysalotosaurus*. The intensity of secondary remodeling is therefore probably not only an indicator of individual age and longevity (e.g. [57], [81]), but also an indicator of maximum body size [82]. This is probably the case in primates (compare e.g. Castanet et al. [83] and Burr [84] with Mulhern & Ubelaker [85], see also Singh et al. [86]), ornithopods (see above), and sauropodomorphs (compare e.g. Klein [32] with Klein & Sander [81]). The comparison of the largest sampled femur of *Dysalotosaurus* (33cm calculated length) with the largest femur of *Dryosaurus* (49cm length; see [52]), which shows much more extensive secondary remodeling, either confirms this assumption, or the latter was indeed individually older than the former [82]. This femur is even larger than the largest preserved, *Dysalotosaurus* femur, which has a calculated length of 38 cm. Together with the observations of increasing secondary remodeling within the ontogenetic stages of *Dysalotosaurus*, the influence of individual age on remodeling intensity is probably most important, but maximum body size might be an additional factor.

Finally, Horner et al. [52] noted that the largest *Dryosaurus* femur was still actively growing, because it lacks an EFS and therefore belonged to a somatically subadult individual. If this is true, then even the largest known individuals of *Dysalotosaurus* were still somatically subadults.

### The Life History of *Dysalotosaurus*

The embryonic or perinatal ontogenetic stage is not preserved in *Dysalotosaurus*, but the longitudinal section of the smallest known femur (Fig. 7D–F) belonging to the early juvenile stage is very distinctive regarding possible behavior of hatchlings. This stage is very similar to the structures described for younger stages of *Orodromeus* and *Troodon* [51], although the pads of calcified cartilage reach naturally much deeper at this early ontogenetic stage than in the sample of *Dysalotosaurus*. It is also in strong contrast to the situation seen in some hadrosaurs [51] where pads of calcified cartilage are not constricted to the preserved epiphysis, but reach through the whole metaphysis into the diaphysis. Endochondral bone is here much rarer and apparently lacks transverse struts crossing the long tubular structures, which consist of connected cartilage canals and marrow processes. In the large nestling of *Maiasaura* [36], thin coatings of endochondral bone are developed along the wall of the marrow processes, but noticeable transverse struts were only observed deeper within the metaphysis. Since large nestlings of *Maiasaura* are here tentatively correlated with the early juvenile stage of femora in *Dysalotosaurus*, the degree of epiphyseal ossification in *Dysalotosaurus* at this stage was strongly different from *Maiasaura* and other hadrosaurs, but similar to *Orodromeus* and *Troodon*, which would implicate precociality in *Dysalotosaurus* hatchlings [51], [87]. Thus, they could follow their parents short after hatching, but experienced rather moderate growth rates compared to the probably semi- to fully altricial hadrosaurs [51]. By the way, the precocial behavior is also assumed for the closest relative of *Dysalotosaurus*, *Dryosaurus altus*, whereas an embryo of the larger taxon *Camptosaurus* was probably altricial similar to *Maiasaura* [88].

Moderate growth rates are visible in the four growth curves of *Dysalotosaurus* (Fig. 20). The early and late juvenile stages of the femur cover the moderately sloping part of the growth curves up to approximately six years of age. The sexually immature stage correlates with the age of six up to ten years. The latter date most likely marks the initiation of sexual maturity and therefore separates the sexually immature members of the *Dysalotosaurus* herd from the sexually mature individuals. This hypothesis was derived from five out of the six sampled large femora belonging to the most mature histological ontogenetic stage observed (see above). A mark or transition (MISM) is visible in these cross sections (Figs. 1B; 4A; 5E–F; 6A–E). This demarcation shows an overall slow-down of bone apposition rates (the usual fast growing zones are weak or absent), which interestingly starts in each of the five concerning femora at almost the same relative position within the cross sections (Figs. 6E; 16; 19; 20). Thus, this mark represents not an individual event, but a real physiological signal indicating an important change in the life history of *Dysalotosaurus*.

The achievement of sexual maturity is the most likely explanation supported by several reasons: (1) This event is commonly combined by a slow-down of growth rate in many other tetrapods (e.g. [7], [57], [62], [89], [90]); (2) The timing of sexual maturity occurs well before somatic maturity as in other dinosaurs (e.g. [32], [57], [62], [75], [91]); (3) This event plots in diagrams with body size versus age almost exactly at the curves point of inflection ([62] but see below); (4) The preservation of medullary bone tissue in a large fibula and a large tibia (Figs. 11; 14A–D), which belong to the group of large individuals in the size-frequency distribution (Fig. 23; Tab. 2); and (5) By correlating the respective value of this mark with femoral size, the mark plots well within the gap between the dominating groups of small and large individuals of the *Dysalotosaurus* herd (Fig. 23).

This gap shows the underrepresentation of individuals and is probably the result of banishment and/or increased mortality of this size class. In recent and at least temporarily gregarious ungulate mammals, young males predominantly suffer increased mortality around the time of sexual maturity, because they are driven out of the herd very early by prime-aged males (e.g. Impala [92]) or they leave on their own (e.g. Kudu [93]). They are then vulnerable to predators and have higher stress levels due to their low rank within bachelor herds. In other species, young males suffer high mortality during their first rut (e.g. bighorn sheep [94] and rhinos [95]). Young females also suffer increased mortality due to inexperience in reproduction, high reproduction costs, and competition with prime-aged females (e.g. red deer [96]). Higher mortality rates resulting from early sexual maturity were also suggested for the tyrannosaur *Albertosaurus* [91]. Thus, the position of the mark right within the gap of the size-frequency distribution (Fig. 23) supports the assumption that it is indeed the Mark of Initial Sexual Maturity (MISM).

The decrease in bone apposition rate observed in the cross sections at the MISM apparently conflicts with its relative position within the growth curves (body mass versus age; Fig. 20), because it is located here within the lower third of the exponential growth phase and growth rate is still accelerating. This is similar to other dinosaur taxa, where the time of sexual maturity is strongly indicated by the occurrence of medullary bone [62] and/or increased midlife mortality [91]. The time of sexual maturity for *Tenontosaurus* (8 years) and *Allosaurus* (10 years) is located, as in *Dysalotosaurus*, within the lower third of the exponential growth phase and not at the curves point of inflection, where growth rate reaches its maximum [62]. In the case of *Tyrannosaurus*, the estimate of 18 years is close to the inflection point, which is similar to *Albertosaurus* (compare [42] with [91]), although the exact time of sexual maturity is probably an upper bound for *Tyrannosaurus* [62].

It is suggested that the phenomenon of contradicting features in *Dysalotosaurus* is an effect of allometric scaling between increasing body mass and increasing body size (including bone apposition), where the ratio would be 8∶1 (compare also Box 3a with 3b in [7]). Furthermore, the scaling effect of body mass is neutralized by plotting a variable representing body size versus age (Fig. 21), where the time of sexual maturity in *Dysalotosaurus* is indeed located almost exactly at the curves point of inflection.

It should also be noted that the MISM is completely absent in all large tibiae and humeri of respective position within the size-frequency distributions. This indicates an only moderate slow down of bone apposition rate, which is probably not visible in elements of slightly lower relative growth rates compared to the rates in femora. Finally, the relative body size at time of sexual maturity compared to maximum known body size in *Dysalotosaurus* is approximately 62 to 64%, which is strikingly similar to the remarked 60% to the recorded maximum size known in *Albertosaurus* [91] and close to the estimated value of 70% in *Barosaurus* [57]. Thus, the apparent contradiction between decelerating bone apposition and accelerating body mass in *Dysalotosaurus* in young sexually mature individuals is treated here as rather insignificant.

The location of the largest sampled femur (SMNS F2 – group two) within the growth curves is well below the estimated asymptote at approximately 16.4 years of age (Fig. 20). Additional features of still active growth are the open vascular canals at the periphery, well vascularized tissue in the external bone wall areas, and the complete absence of an EFS. The third largest known femur (R12277) is also located below the asymptotic level of the growth curves indicating that this individual has also not reached somatic maturity. The subsequent sampling of the largest known femur (MB.R.Ig374; similar to the specimen used to calculate maximum body size and mass (MB.R.2144)) also revealed still active growth. The absence of EFS in a much larger femur of the closely related taxon *Dryosaurus altus* [52] suggests that this species obviously grew to larger body sizes than *Dysalotosaurus* and that both taxa most likely experienced indeterminate growth as Chinsamy [18] already suggested.

Many of the *Dysalotosaurus* individuals could be reproductively active for more than five years, but none of them obviously reached somatic maturity. *Dysalotosaurus* was highly vulnerable to most of the contemporaneous predators due to its relative small body size and the lack of any defensive structures (as in *Kentrosaurus*). This may be a reason, why sexual maturity was delayed until the ninth year of life. The cost of reproduction was too high for small individuals due to high vulnerability to predation.

Another factor for the high mortality rate around time of sexual maturity and, especially, the prolonged exponential growth phase in sexually mature individuals might be intraspecific competition within a herd. Larger/stronger individuals surely had a more dominant role within the herd and a better chance for reproduction than smaller/weaker individuals. Fast and extended indeterminate growth could therefore be regarded as a survival advantage for *Dysalotosaurus*.

### Implications for the Growth Pattern in Other Ornithopods

Like in some other small ornithopods and many sauropods [18], [37], [48], [52], [55], [56], [57], [76], [80], [97] *Dysalotosaurus* exhibits a growth pattern, where annuli/LAGs as representatives of a zonal bone tissue are rather scarce, completely absent, or are replaced by less obvious growth cycles. On the other hand, large ornithopods, other ornithischians, prosauropods, and all theropods more derived than *Herrerasaurus* (see [6], [18]) show a relative consistent growth pattern with annuli/LAGs representing the usual kind of growth cycles (e.g. [25], [27], [28], [32], [35], [36], [38], [68], [75]).

Klevezal [22] has found a relationship between the abundance and uniformity of annuli/LAGs and environmental conditions in recent mammalian populations, which could partially explain the sorting of dinosaurs into such multiform groups. Populations inhabiting regions with strong seasonality consist mainly of individuals with distinct and weakly variable annuli/LAGs in their recording structures (e.g. bone microstructure), which is mostly a two-phase annual rhythm. In contrast, populations of the same species, inhabiting regions with moderate conditions, exhibit mostly weakly-developed annuli/LAGs and a higher variability in number (poly-phase annual rhythm). However, exceptions always occur. So, although it is likely that a single fossil specimen represents the usual growth pattern of its population, it is also possible that it represents the anomalous minority. An unusual growth pattern found in a single specimen should therefore be treated with caution (see [48], [52], [98]).

The regular development of annuli/LAGs in highly seasonal regions is advantageous compared to irregular cyclicity, because the former is synchronized to the seasonal changes of environmental conditions. Irregular or asynchronous growth is disadvantageous in strongly seasonal regions, because growth phases reaching into harsh times cost naturally more energy than arrested growth. Poly-phase growing individuals have therefore to fit their growth regime to the seasonal conditions or die. In less seasonal regions, it does not matter, which growth regime an individual possess, because the effects on its energy balance is not so disadvantageous and the variability of growth patterns in the population is therefore much higher [22].

The results for *Dysalotosaurus* have shown that the abundance and development of annuli/LAGs depends either on relative growth rate (annuli/LAGs in faster growing femora are less abundant) and environmental conditions (by far not all growth cycles are completed by an annulus/LAG). For the Tendaguru region with its reconstructed seasonal change of humidity [15], long droughts would be such harsh times accompanied by a shortage of food and water. This is also indicated by the depositional area of the Tendaguru Beds, which are very unlikely to be the usual habitat for the preserved dinosaurs [17].

LAGs are obviously more common in ornithopods than previously thought [98] and completely azonal bone is rather unlikely (in contrast to e.g. [6], [18], [48]). LAGs occur in *Orodromeus*, *Dysalotosaurus*, *Tenontosaurus*, and *Maiasaura* at first in the late juvenile stage (this study and [34], [36], [52]). In *Dysalotosaurus*, LAGs are very rare and close to the periphery at this stage (except in humeri). The first LAG in *Tenontosaurus* is also not consistently developed in all specimens and is sometimes substituted by a band of differing oriented collagen fibrils [34]. In *Dryosaurus altus*, LAGs were found in all three subadult femora, but at non-overlapping relative positions indicating at least three different growth cycles for the two smaller specimens and up to 15, if one includes the largest femur and calculates the number of LAGs by back counting [52]. If *Dryosaurus* is indeed similar to *Dysalotosaurus* in its growth pattern, which is implicated by a similar vascularization pattern and the absence of an EFS, then the number of developed LAGs would be still rare in the large femora of *Dryosaurus*. In *Dysalotosaurus*, ten out of 14 femora (excluding the juvenile stages) bear one (in one case two) LAG or annulus (Fig. 5A–B), respectively (in Tab. 1 six out of nine, excluding the femora not usable for the age calculations), but these annuli/LAGs represent at least three to four non-overlapping positions, which confirms a very inconsistent and highly variable growth pattern. It is therefore possible that Chinsamy [18] sampled specimens, where LAGs are not developed among the other growth cycles.

*Orodromeus* differs from both *Dysalotosaurus* and *Dryosaurus* by its lower overall growth rate (see above) and the presence of an EFS in the largest individuals [52]. Another difference is the quiet consistent development of LAGs in the tibiae and femora of subadult and adult individuals. This could be the consequence of overall lower growth rates in *Orodromeus* [52]. The development of LAGs is more likely, because the seasonal slow-down in growth starts from an already lower level than in *Dysalotosaurus* and *Dryosaurus*. However, *Orodromeus* seems to be rather an exception among small to medium-sized ornithopods regarding its growth pattern, although LAGs and annuli were recently also found in small ornithopods from high latitudes [98].

The age of *Orodromeus* at the beginning of somatic maturity is estimated by Horner et al. [52] at five to six years. This is relatively short for a dinosaur of this size, because other small dinosaur taxa reached ages of at least nine and eight to 18 years, respectively [27], [75]. Scheetz [53] described four additional bands of highly birefringent bone tissue alternating with weakly birefringent darker bands in a juvenile femur of *Orodromeus* (see also figure 2C in [52]). At a first glance, it has some similarities to the alternation of fast and slow growing zones in *Dysalotosaurus*, although such a suggestion should be treated with caution. If these bands are indeed annual cycles, than the age of *Orodromeus* would be about ten years at time of reaching somatic maturity. This would fit much better to the estimated ages of other small dinosaurs.

**Figure 24:**
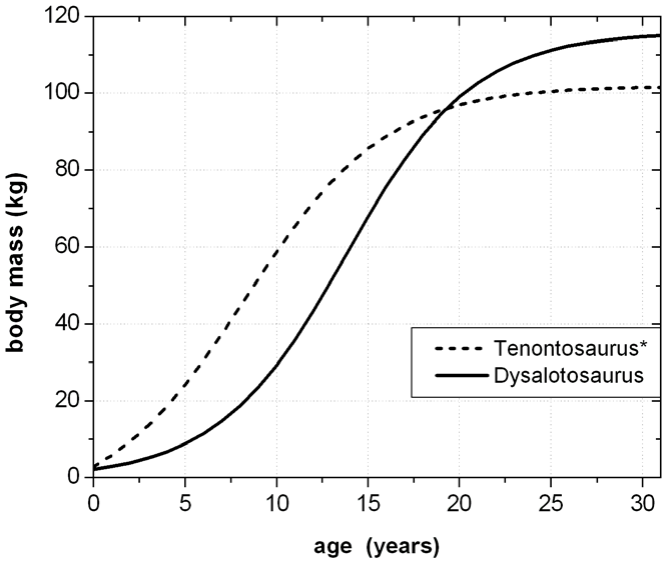
Comparison of growth curves of *Tenontosaurus tilletti* (derived from table 2 in [**62**]) and *Dysalotosaurus lettowvorbecki*. *Note that the maximum body mass of *Tenontosaurus* is app. ten times higher than in *Dysalotosaurus*. Thus, for a better comparison, the body mass values of *Tenontosaurus* were divided by 10 and then used for the growth curve calculation.

The three larger ornithopods *Tenontosaurus*, *Maiasaura*, and *Hypacrosaurus* developed much higher numbers of LAGs in the subadult and adult stages than *Dysalotosaurus* and *Dryosaurus* before reaching somatic maturity [34], [36], [38]. They experienced very high growth rates during the juvenile stages (e.g. [36]), as the growth curve of *Tenontosaurus* also shows in comparison to the averaged growth curve of *Dysalotosaurus* (Fig. 24). Thus, all three large ornithopods had higher initial and juvenile growth rates and reached their asymptotic growth plateau relatively earlier than *Dysalotosaurus* and most of the other small ornithopods.

By using the mentioned relationship between strength of seasonality of environmental factors and occurrence and uniformity of annuli/LAGs [22], the abundance of numerous annuli/LAGs in subadults and adults of larger ornithopod taxa would indicate higher seasonal stress than in the smaller *Dysalotosaurus* and *Dryosaurus*. Another example is the absence of annuli/LAGs in the small Proctor Lake ornithopod compared to their occurrence in a large hadrosaur of the same locality [55]. The zonation in just a single femur of *Gasparinisaura* (assuming that the others lack it [54]) probably represents similar intraspecific variation of cyclical growth patterns than in *Dysalotosaurus*, although LAGs are even completely unknown.

Thus, many small ornithopods had probably less seasonal environmental stress than large ornithopods and different growth patterns had existed in large and small taxa, respectively. Two reasons are proposed for these differences:

(1) *Food demands and migration:* Small ornithopods were predominantly selective low-browsers [99] and probably not able for supra-regional migration [100]. They needed less absolute amounts of food than large ornithopods, which would also have a weaker effect on their growth rates during dry (or cold) seasons than in large taxa. The ability to alternative nutrition, such as insectivory [20], [53], might also have played a role. Large ornithopods cleared their local habitat of food much faster than small ornithopods, not only, because of their higher absolute food demands, but also due to their much more effective chewing ability (e.g. [101], [102]) and their assumed gregarious behavior (e.g. [101], [103], [104], [105]). For many of them, migration was therefore essential to survive and this meant additional seasonal stress. Furthermore, some small ornithopods were probably able to endure bad times by specialized adaptations, such as the fossorial *Oryctodromeus* ([106], see also [107]), to which larger ornithopods were unable to do so [100]. However, the recent discovery of annuli/LAGs in the small polar ornithopods from southern Australia ([98] in contrast to [48]) demonstrates that even low seasonal liability did not prevent them from the severe polar winters of their habitat so that they had to stop growth for saving vital energy during the dark season.

In conclusion, higher food demands and seasonal migration of large ornithopods could be one reason for the much more consistent development of annuli/LAGs in their long bones compared to small ornithopods. Exceptions may be the ornithopods *Telmatosaurus* and *Zalmoxes*, which are treated as secondarily downsized taxa due to their restricted island habitat [108].

(2) *Breeding strategy and courtship/rut: Dysalotosaurus*, *Orodromeus*, and other smaller ornithopods were probably precocial as hatchlings (see above; [51], [55]), whereas hadrosaurs were mainly altricial [36], [51], [104]. Parents of precocial offspring only have to care for the eggs and have to protect and lead the young within the herd. The latter task could also be managed by other members of the herd, so that the individual stress of single parents was even lower. Altricial behavior, in contrast, means the possibility of extraordinary high juvenile growth rates on the one hand, but also more stress for the caring parents on the other hand. Parents of an altricial offspring have to feed their young and have to protect them against other members of the colony as well as against carnivores of all sizes. Colonial nesting is also a stress factor in itself, because many individuals are concentrated in a comparatively small area [104]. In addition, at least the sexually dimorphic lambeosaurine hadrosaurs could have had a seasonal rut or courtship [101], which also would mean higher seasonal stress for sexually mature individuals. Thus, the large hadrosaurs likely suffered much more stress as sexually mature individuals, but their altricial behavior equalized this disadvantage due to the ability to outgrow other dinosaurs as juveniles, especially all contemporaneous theropods [49]. The growth pattern of *Tenontosaurus* ([34], [62]; Fig. 24) is similar to hadrosaurs, so that altricial behavior can be assumed as well. Thus, altricial behavior was probably one of the key strategies within Ornithopoda to become large in a short time and the resulting growth pattern (higher juvenile growth rates, early sexual and somatic maturity compared to small ornithopods, consistent development of annuli/LAGs) reflects this seasonally much more stressful strategy.

It is important to note that the remarks on the reasons for different growth patterns in ornithopods are tentative hypotheses. The variability of growth patterns, especially in smaller ornithopods, is striking and ontogenetic histological studies of more taxa are urgently needed to strengthen or disprove them. Nevertheless, the occurrence and/or consistency of annuli/LAGs in ornithopods is dependent on a mixture of absolute growth rates (which depends on maximum body size), relative growth rates (depends on the sampled skeletal element and its ontogenetic stage), the degree of seasonality of the respective habitat, and the liability of the taxon to seasonal effects including temperature, humidity, food supply, migration, and behavior (e.g. precocial or altricial breeding strategy). Phylogeny plays a rather unimportant role, as already indicated by Werning [34].

### Conclusions

The large amount of specimens, representing a wide range of ontogenetic stages, offered the unique opportunity to learn more about the modes and reasons of variation in bone tissues and allowed insight into the growth pattern and life history of the ornithopod dinosaur *Dysalotosaurus*. For this purpose, up to 70 individual bones were sampled, comprising femora, tibiae, humeri, fibulae, and prepubic processes.

Variation within the bone tissue was mainly found between different skeletal elements and between different units of single cross sections. The former is the result of different relative growth rates, which are dependent on the individual size of a certain element and its degree of utilization within the skeleton. Skeletal elements with a large absolute size, with main weight bearing functions, and elements intensively used for movements (e.g. for locomotion) experience higher relative growth rates than other elements. Some elements have of course combined these characters, which explain the highest growth rates in the femur for instance. Accordingly, the only predictable model on the occurrence of annuli/LAGs in *Dysalotosaurus* is their increasing abundance in skeletal elements with lower relative growth rate. The number of growth cycles naturally increases during ontogeny, but this definitely is not the case for annuli/LAGs. The extraordinary variation in the development of annuli/LAGs in *Dysalotosaurus* eliminates prediction of their existence and relative number in skeletal elements of different ontogenetic stages.

Intra-cortical variation in bone tissue is mainly the result of osseous drift and variation in bone wall thickness during growth. The relationship between osseous drift, bone wall thickness, bone tissue variation, and resulting relative growth rates, can now be better defined:

- A long bone with a bended long axis experiences osseous drift from the convex to the concave side of this long axis.
- Relative growth rates, derived from the organizational degree and the density of vascular canals, are lower on the convex side of the bended long axis and higher on its concave side.
- Growth rates are also relatively higher in thicker cross sectional units than in thinner units.
- Variation in bone tissue within a cross section decreases the more consistent and round the transverse shape of a bone is. A shaft with a triangular transverse outline contains more variation than a shaft with a circular transverse outline.
- In the case of partial sampling of a bended long bone, the part with the best potential record of ordinary bone tissue and growth cycles is the flat wall on its concave side.

The bone histology of *Dysalotosaurus* is most similar to *Dryosaurus altus* in respect of ontogenetic stages, rarity of annuli/LAGs, variation of bone tissues, low degree of secondary remodeling, and the absence of an External Fundamental System. This confirms the close relationship and a similar growth pattern and general life style of these taxa.

A new type of growth cycles was used to reconstruct the life history of *Dysalotosaurus*, despite the scarcity and variability of annuli/LAGs. Growth curves of femora (derived from this alternation of fast and slow growing zones) revealed that *Dysalotosaurus* grew with a moderate rate in its juvenile stage until approximately six years of age, experienced accelerated growth during its sexually immature stage until reaching sexual maturity at approximately ten years of age, and had its exponential growth phase as sexually mature individual until the 14^th^ year of life, where the maximum growth rate was reached. Afterwards, the growth rate decelerated and might have reached asymptotic growth well after 20 years. However, most likely none of the members of the *Dysalotosaurus* herd reached the growth plateau of somatic maturity.

The group of large individuals within the size-frequency distribution obviously consists of sexually mature individuals, because medullary bone was found in a tibia and a fibula of this size range. The time of initial sexual maturity was discovered as a transitional mark (MISM) in five large femora representing a slight slow-down of bone apposition rates.

Indeterminate growth, combined with delayed sexual maturity, is assumed to represent the optimal growth strategy of *Dysalotosaurus* to withstand intra-specific competition and its high liability for predation.

The results of the bone histological study of *Dysalotosaurus* were finally combined with a relationship between abundance and consistency of annuli/LAGs in recent mammals and their respective seasonal environment. Smaller species of ornithopods are less exposed to seasonal effects than the large species mainly based on differences in food demands, growth rates, and breeding strategy. In fact, the achievement of large size within Ornithopoda was probably linked to a change in breeding strategy from precocial to altricial behavior.

## Materials and Methods

The key literature for an introduction into bone histology, where also the here used terms are explained, comprises Castanet et al. [5], Chinsamy-Turan [6], Erickson [7], Francillon-Vieillot et al. [8], Klevezal [22], and Ricqles et al. [10].

The sections used by Chinsamy [18] could not be re-examined, so that this study is completely based upon newly produced thin sections.

30 femora, 12 tibiae, 13 humeri, seven fibulae, and eight prepubic processes were sampled, but not all of the obtained thin sections were well preserved. Thus, 11 femora, two tibiae, and four humeri were inappropriate to be considered for measurements and correlations and are therefore also not included in the Tables 1, 2, and 3.

### Location and Production of Thin Sections

**Figure 25:**
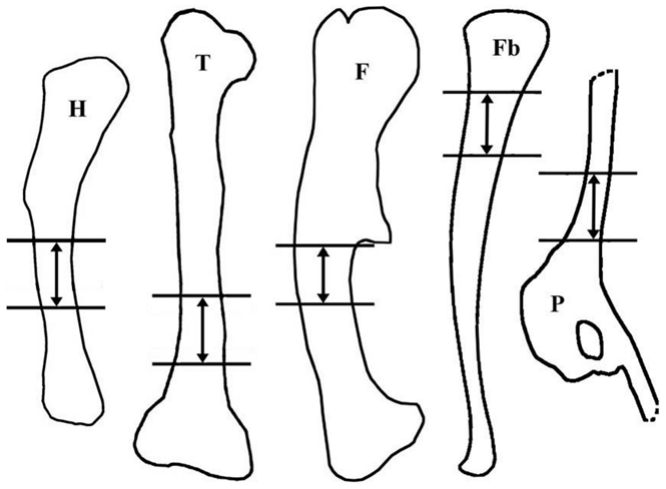
Intervals of cutting levels in the sampled elements. Abbr.: F – Femur (lateral view); Fb – Fibula (lateral view); H – Humerus (anteromedial view); P – Pubis (lateral view); T – Tibia (posterior view). Elements are not scaled.

The bones used for thin sectioning were loaned from the collections of the Geowissenschaftliches Zentrum, University of Göttingen (GZG), the Staatliches Museum für Naturkunde, Stuttgart (SMNS), and the Institut und Museum für Geologie und Paläontologie, University of Tübingen (GPIT). Measurements of additional specimens were also made in the Museum für Naturkunde, Berlin (MB) and the Natural History Museum, London (R/NHMUK). All sampled bones (femora, tibiae, humeri, fibulae, and pubii) where already broken, lacking either the distal or proximal ends. In case of the femora, it was also possible to use isolated shafts, because the distal beginning of the fourth trochanter or the medial depression helped to clarify its orientation and the best position for the thin section. The prepubic process of the pubis represents the only non-long bone element and was chosen to highlight further variability within the skeleton of *Dysalotosaurus*. It is important to note that it was impossible to take thin sections from a standard level, because only incomplete specimens were used. Furthermore, it was aimed to cause as less damage as possible, so that most of the cuts were carried out close to broken surfaces. Thus, the sections are standardized to a single interval along the bone shaft and not to a single level (Fig. 25). Distinct processes or expansions helped to verify the relative position of the section.

The bones were cross cut with a diamond powder disk on a precision saw. Due to the brittle nature of many bones, they were temporally embedded in acetone dissolvable two-component epoxy-resin (Technovit 5071) during the sawing process. By the following well known process of grinding and cutting (see e.g. [9], [22], [109]), the produced thin section got a final thickness of approximately 100 µm.

### Sorting of Thin Sections

The description of bone tissue types and structures found in the sampled bones of *Dysalotosaurus* generalizes the observations for each of the elements. Some cross sectional units have very special and recognizable features, which helped to orientate them even without the actual bone. Distances are only measured along the anteroposterior axis or the mediolateral axis (see Tables 1, 2, and 3). All steps beyond the description, which incorporates the count and correlation of growth cycles, were only done with femora, tibiae, and humeri. The other sampled elements were too close to the metaphysis (fibulae) or they had a too thin periosteal bone wall (pubii) to gain enough quantitative information.

All thin sections with growth cycles were sketched using Adobe Photoshop 7.0 software. Due to the large error in taking standardized thin sections, it was impossible to simply superimpose the sketches of different ontogenetic stages of a sampled element to get a complete record of all growth cycles from the smallest to the largest specimen. Thus, thin sections of femora were sorted into four groups and the sections of the tibiae into two groups depending on cutting level and cross sectional shape. Humeri were not sorted due to the relative constancy of the outer cross sectional shape (Tables 1, 2, and 3).

### Conversion of Growth Cycles into Absolute Age Estimates

The basic assumption is the annual character of the present growth cycles (see above). It was the goal to correlate the cycles of all cross sections of one group of a single skeletal element, to count the final number of cycles, and to equalize them into years. Superimposition of sketches did not lead to a good correlation of growth cycles due to variation in cross sectional shape and the course and distances of growth cycles to each other. Hence, another way was chosen to get a correlation, which was also carried out by using Adobe Photoshop 7.0 software.

The end of each growth cycle was marked in the sketches by a permanent line. A standard location within the cross sections, which usually revealed the best record of growth cycles, was determined for femora, tibiae, and humeri respectively. In tibiae, two fitting locations were found and the final growth cycle values were then averaged.

**Figure 26:**
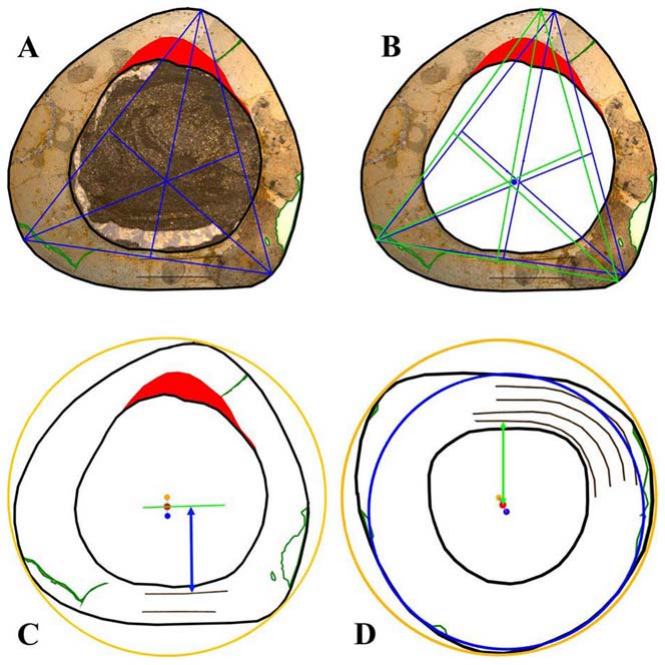
Sketches showing important steps to gain a standardized midpoint in cross sections for the measurement of distances between this midpoint and the external border of each growth cycle. A–C: Late juvenile femur GZG.V 6590 28: A – First triangle with its vertices on the utmost extremities of each corner; B – Second triangle with vertices extrapolated from the respective opposing straight walls. The blue point in the centre is the midpoint from both triangles; C – The final midpoint of the cross section is derived from the blue midpoint of the triangles and the orange midpoint of the sketched circle. The green line lies parallel to the course of the growth cycles and the distances (e.g. blue double arrow) are then measured perpendicular to the cycles in the posterolateral part of the posterior wall. D – Late juvenile tibia GPIT/RE/3724: The midpoints of an inner and an outer circle (blue and orange, respectively) are used to get the final midpoint (red) for measuring the growth cycle distances. All sketches are not scaled, but consistently oriented with the anterior direction at the top and the medial direction at the left. The red area in A–C represents the anterior CCCB-wedge. Lines in green mark damage of the cross sections.

The first step towards the correlation of cycles was the definition of an unambiguous and repeatable midpoint for every used cross section (Fig. 26). Femoral cross sections mostly have a triangular shape, so that two types of geometric triangles were generated. The vertices of the first triangle were set on the utmost extremity of each of the three corners of the cross section (Fig. 26A). The vertices of the second triangle were generated by three straight lines, which were placed on the external edge of the three straight walls. Each line was then graphically shifted onto the utmost extremity of the opposing corner in the cross section and the respective vertex was set. The midpoint of both triangles was generated, but the midpoints of both triangles did not coincide in most cases. The midpoint of a straight line, drawn between both triangle midpoints, was therefore defined (Fig. 26B). To minimize possible error, a circle was additionally drawn as large as possible to fit right on the outer contour of the femoral cross section. Another straight line was created between the midpoint of this circle and the combined midpoint of both triangles, so that the actual midpoint of the whole femoral cross section was the midpoint of this line (Fig. 26C).

The cross sectional shape of tibiae and humeri were much more oval in shape. Here, the midpoints of two circles were used to determine the midpoint of the cross section. One circle was graphically scaled down as small as possible to enclose the cross section externally and just tangent the outer edge. The second circle was scaled up as large as possible to tangent the outer cross sectional edge internally. The midpoint of a straight line, which was drawn between the two obtained circle midpoints, was then determined as the midpoint of the cross section (Fig. 26D).

During the next step, the distance between the cross sectional midpoint and each of the recorded growth cycles was measured and transformed into partial percentages of the distance between the midpoint and the external cross sectional edge. The reference measurement for each of the cross sections, representing 100% from midpoint to external edge, was already measured before at the respective sampled specimen. Since not the same reference measurement could be taken from each of the femora, tibiae, and humeri, regression equations were calculated with Microsoft Office Excel 2007 software to get the allometric relationships for these distances. In the end, all reference measurements were transformed into diaphyseal circumference and distal mediolateral width in femora, distal mediolateral width for tibiae, and mediolateral width at the deltopectoral crest for humeri (Tables 1, 2, and 3). The data for the allometric calculation was taken from the measurement dataset of complete specimens of these long bones (Table S1).

It is important to note that each measurement from the cross sectional midpoint to a growth cycle was taken perpendicular to the course of the latter. In all femora, the best growth record was preserved in the lateral part of the posterior wall, close to the posterolateral corner. A straight line was drawn from the midpoint parallel to the course of the growth cycles and the measurement was then taken laterally from the midpoint and perpendicular to the course of the growth cycles (Fig. 26C). In all tibiae and humeri, such an additional line was not necessary and all measurements were directly taken from the midpoint. The best growth record in tibiae was preserved in anterior and medial direction and in the humeri in anterior direction only.

A special cycle, observed in five large femora and marking initial sexual maturity (MISM), was measured in the same way as the growth cycles.

All measured percentages of growth cycles were then transformed, in a third step, into partial values of the reference distance of the respective cross section (representing 100%) and recorded in an Excel file. The values of each cross section were sorted in their respective group, one in humeri, two in tibiae, and four in femora. The following correlation of growth cycles was therefore done only within a single group. The still uncorrelated growth cycles of each group were related to age in years. A diagram was then created, were the x-axis represents age and the y-axis partial reference values of the growth cycles of each cross section of this group. The correlation of growth cycles to age in years started by fitting the lowest known value to an age of one year. This could be done, because the respective value was derived from the smallest sampled specimen (Tab. 1), where the corresponding growth cycle was found at the outer edge of the completely unremodelled and unresorbed bone wall (Fig. 8A–B). The distance of successive growth cycles of the other cross sections in the dataset as well as the diagram revealed the general distance of values between two successive years. First, all values of a single cross section were shifted, so that the smallest value of a cross section fit onto a value of another one. In this way, the values of every single section of this group were fitted to get a single curve in the diagram, where possible outliers are minimized. It occurred especially in large or strongly obscured cross sections that the successive growth cycle values could be separated, because the large distance between them could be filled by successive values of other sections. The MISM was separately signed into the diagrams of two groups of femora.

### Calculation of Body Mass

Two out of four groups of sampled femora were chosen to convert their age related growth cycles into body mass estimates. The samples of the other two groups are not appropriate, because their location within the shaft is either too proximal or too distal, and their small number of recorded growth cycles only covers three to four years. In contrast, growth cycles of several samples in femoral group one and two were often placed within the same year of age during correlation. In this case, the average of all values of this year was used as the basis for the body mass calculation.

Two methods of calculating body mass by skeletal elements were considered. The first method was derived by Anderson et al. [64] by using the combined humeral and femoral shaft circumference to calculate body mass in quadruped animals. For biped animals, only the femoral shaft circumference was necessary. The following equation was therefore used for *Dysalotosaurus* femora, W = 0.16 C_F_^2.73^, where W is the weight and C_F_ is the circumference of the femur.

The accuracy of this method was recently doubted [110]. However, the conventional model predicts the body mass of small to medium-sized animals much better than the proposed alternative [111]. It is also more reliable to the natural variability of body mass in different size categories than the proposed non-linear alternative [111]. Thus, it is assumed that the method of Anderson et al. [64] used here is still the best model to predict the body mass in the rather small-bodied dinosaur *Dysalotosaurus*.

The second method was derived by Erickson & Tumanova [27] known as Developmental Mass Extrapolation (DME). The basis for this body mass calculation, which emphasizes the effect of ontogeny on mass increase, is the assumption that the approximately third power of femoral length corresponds to body mass in *Alligator* (data in [112]) and the California Gull (data in [113]). Both species represent members of outgroups of non-avian dinosaurs (Extant Phylogenetic Bracket [114]), so that the ratio of femoral length to body mass could also be used for *Dysalotosaurus*. This was also done for the respective values of the MISM.

### Establishing the Growth Curve

To compare the life history of *Dysalotosaurus* to other dinosaurs and recent animals, a type of growth curve had been chosen, which was already used by Erickson et al. [77].

The calculated body mass of the averaged growth cycles was therefore plotted against their respective age in years. The equation y = a/(1+exp (b * (x+c)))+d describes the sigmoidal course of this type of growth curve (y = body mass; x = age in years; a = largest known body mass; b, c, d = parameters to fit). The variable *a* was derived from the largest known femur with a calculated body mass of 115.3 kilograms. Only the secured growth cycle values were integrated and all unsecured values, including the values externally to the MISM, were excluded. The latter values were entered afterwards into the curves to evaluate their significance and possible age correlation. The MISM itself was included with the corresponding age of 9.5 years in femur group one and 10.5 years in femur group two. A total of four curves were created, including the calculated body masses by the methods of Anderson et al. [64] and Erickson & Tumanova [27] for femoral group one and two, respectively. The dataset was entered into the software Microcal Origin and the non-linear curve fit function (basing on least-square regression analysis) was performed using the equation mentioned above.

### Growth Rates and Age/Size Frequency Distribution

To get yearly and daily growth rates, the calculated yearly body masses were derived by using the sigmoidal equations and the four parameters of each of the four growth curves. One version corresponds to the growth rate in a recent year (365 days) and the second version corresponds to a year in the Late Jurassic (Kimmeridge, 150 million years ago), which contained approximately 377.76 days [27], [115]. The maximum growth rate per day, calculated in gram, was then plotted into the diagram of Erickson et al. [77].

The final step was the combination of the absolute age estimates with the size frequency distribution of all femora (Fig. 23), so that one can assign a certain position within this distribution to a certain age. First, the allometric relationships for the femoral distal mediolateral width and femoral circumference were determined by combining the values of all measured specimens with the sectioned samples. The allometric relationship for the femoral circumference and length was obtained from the measured specimens with both distances preserved (Table S1). Second, the calculation of age for all femora was carried out by conversion of the sigmoidal equation to x (age in years), which resulted in the following equation: *x = ln ((a/(y−d)−1)/b+c* (y = body mass calculated by either the method of [64] or [27]; the parameters a, b, c, d were derived from each of the four growth curves). The obtained ages of the separately calculated versions for both femoral groups were averaged for the dataset derived from the Anderson et al. [64] body mass calculation and for the dataset derived from the Erickson & Tumanova [27] body mass calculation. These average estimates were then correlated with the circumferences and distal mediolateral widths of the femora. Thus, every single value of both measured distances can now be assigned to a specific age (see Tab. 1).

## Supporting Information

Figure S1 **Detail of cross section of tibia SMNS T 13, under polarized light; Anterolateral unit internally; Marrow cavity at top left.** The original vascularization is obviously altered by postmortem dissolution of bone tissue. Former primary osteons are lost during this process and the vascular canals are widened. Scale bar = 500 µm.
(TIF)

Table S1 **List of all specimens and measured data of humeri, tibiae, and femora, which were used for the allometric calculation of the reference values necessary for the correlation of growth cycles in the sampled specimens.**
(DOC)

Text S1 **This text comprises a more comprehensive description of the thin sections of all five skeletal elements of** ***Dysalotosaurus*** **and additionally includes notes on the modes of preservation of the bone microstructure as well as on the occurrence and distribution of osteocyte lacunae and Sharpey's fibers.**
(DOC)
