# Supplementary figures and images for: Bone Histology in Dysalotosaurus lettowvorbecki (Ornithischia: Iguanodontia) – Variation, Growth, and Implications
Source: PLoS One. 2012 Jan 6;7(1):e29958. doi: 10.1371/journal.pone.0029958 (PMC3253128; doi:10.1371/journal.pone.0029958)

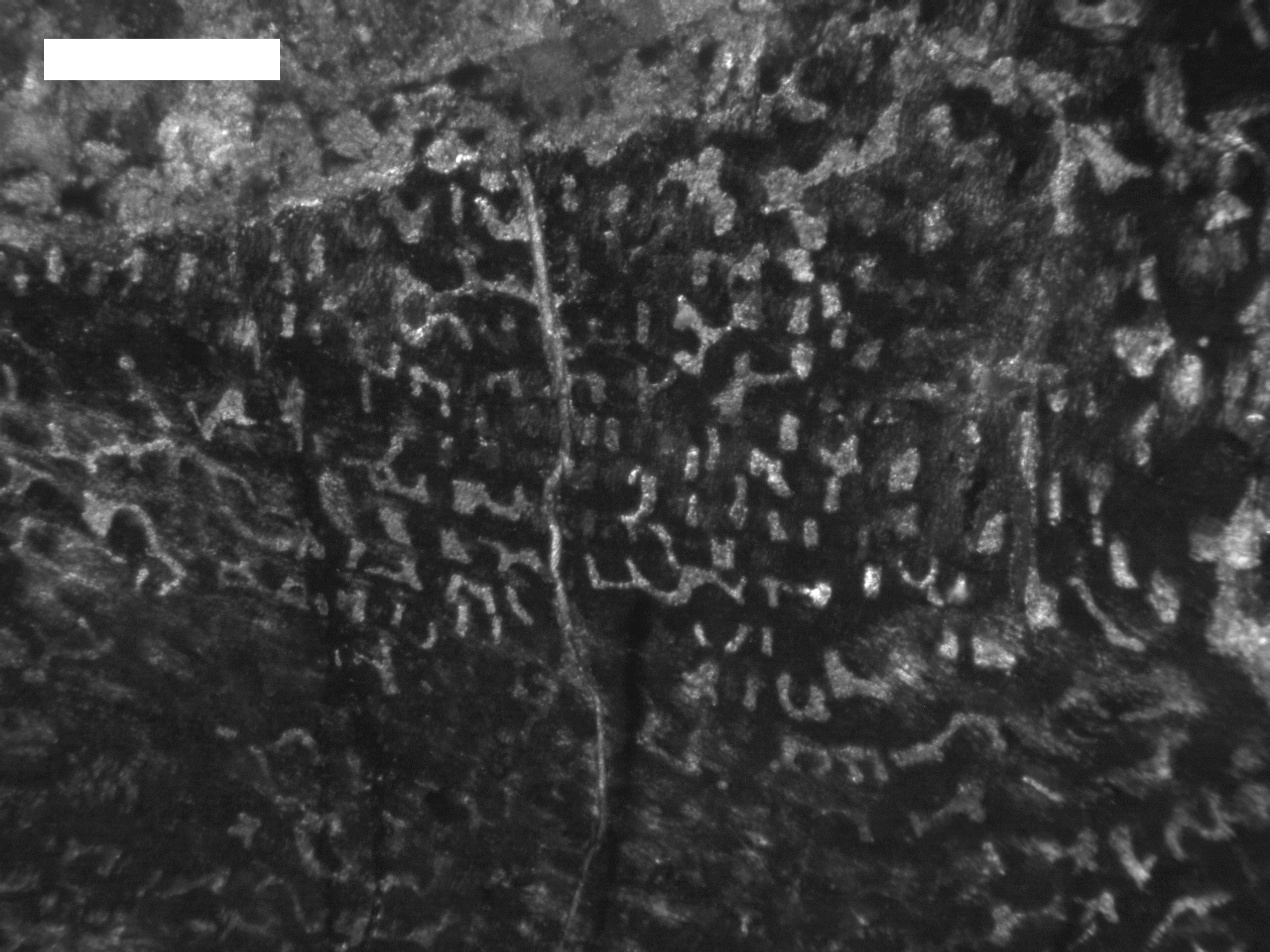

Supplement: Figure S1 — Detail of cross section of tibia SMNS T 13, under polarized light; Anterolateral unit internally; Marrow cavity at top left. The original vascularization is obviously altered by postmortem dissolution of bone tissue. Former primary osteons are lost during this process and the vascular canals are widened. Scale bar = 500 µm. (TIF) [file pone.0029958.s001.tif]
