# Supplementary material for: Bone Histology in Dysalotosaurus lettowvorbecki (Ornithischia: Iguanodontia) – Variation, Growth, and Implications
Source: PLoS One. 2012 Jan 6;7(1):e29958. doi: 10.1371/journal.pone.0029958 (PMC3253128; doi:10.1371/journal.pone.0029958)
Supplement: Table S1 — List of all specimens and measured data of humeri, tibiae, and femora, which were used for the allometric calculation of the reference values necessary for the correlation of growth cycles in the sampled specimens. (DOC) [file pone.0029958.s002.doc]

Table S1: Measured values of more complete humeri, femora, and tibiae, which were used to get consistent reference values for the sampled specimens by calculating allometric relationships.

| Humeri → |  |  |  |  | Femora → |  |  |  |  | Tibiae → |  |  |
| --- | --- | --- | --- | --- | --- | --- | --- | --- | --- | --- | --- | --- |
| Labels | TL | PMW | MWDC | DMW | Labels | TL | MTLS | DMW | CM | Labels | TL | DMW |
| MB.R.1711r | 96.6 | ― | 15.3 | 19.1 | MB.R.2511r | 330 | 42 | 79 | 122 | MB.R.2510l | 174 | 39.3 |
| MB.R.5092l | 157 | 38.6 | 22 | 30.7 | MB.R.2517l | 188 | 23.7 | 42.2 | 67 | MB.R.2512r | 340 | 89 |
| MB.R.5091r | ― | 37.2 | 23 | 30.4 | MB.R.2519l | 119 | 15.5 | 28 | 45 | MB.R.2513r | 325 | 79.6 |
| GPIT/RE/3450r | 68.7 | 16.1 | 10.5 | 12.4 | MB.R.3299l | ― | 22.2 | 44.1 | ― | MB.R.2514r | 300 | 76.4 |
| GPIT/RE/3948l | 70.8 | 17.4 | 10.9 | 13.3 | MB.R.3302l | 300 | ― | 65.7 | ― | MB.R.2515r | 265 | 61.5 |
| GPIT/RE/4013r | 82.1 | 19.3 | 12.3 | ― | MB.R.2144l | ― | 51 | 90 | ― | MB.R.2516r | 200 | 49 |
| GPIT/RE/4167l | 66.7 | ― | 9.6 | 12.2 | MB.R.2508r | ― | ― | 79 | 121 | MB.R.2523l | 165 | 38.2 |
| GPIT/RE/5114l | 77.9 | 18.1 | 11.6 | 14.8 | MB.R.2507l | 306 | 37.9 | 71.4 | 110 | MB.R.2522r | ― | ― |
| GPIT/RE/5731r | 104.7 | 28.3 | 16.5 | 19.9 | MB.R.2506l | 298 | 38.6 | 72.8 | 113 | MB.R.2520r | ― | 28.9 |
| GPIT/RE/6543l | 83 | 21.1 | 13 | 16 | MB.R.1502l | 320 | 39.8 | 74.5 | 111 | MB.R.1709l | 116 | 25.3 |
| GPIT/RE/3448r | 143 | 39 | 23.5 | 28.6 | MB.R.2503r | 310 | ― | 69 | ― | MB.R.5101.1r | 325 | 75.8 |
| SMNSoN1r | 102.2 | 26.2 | 15.3 | 20.9 | MB.R.2500r | ― | ― | 53.5 | ― | MB.R.5102l | ― | 77.8 |
| SMNSoN2r | 113.8 | 29.5 | 15.7 | 21.6 | MB.R.2509r | 185 | ― | 40 | 65 | R12279r | 370 | 89.5 |
| SMNSoN3r | 173 | 47.1 | 25.7 | 33.6 | MB.R.2501r | 225 | ― | 49.2 | ― | R8351r | ― | ― |
| SMNSoN4l | 170 | ― | 26 | 36.7 | MB.R.5099r | ― | 37.5 | 65.2 | 117 | GZG.V.6613r | ― | ― |
|  |  |  |  |  | MB.R.5100l | 280 | 37 | 64.3 | ― | SMNSoN1r | ― | 36.7 |
|  |  |  |  |  | R12278r | ― | 40.6 | 77.8 | ― | SMNSoN2l | 173 | 38.1 |
|  |  |  |  |  | R12277r | 350 | 47.5 | 84 | ― | SMNSoN3r | 166 | 35.9 |
|  |  |  |  |  | R6861r | 198 | ― | 44 | ― | SMNSoN4r | 296 | 69.2 |
|  |  |  |  |  | GZG.V.6273l | 295 | 38.9 | 71.9 | ― | SMNSoN5l | ― | 70.4 |
|  |  |  |  |  | GZG.V.6277l | ― | ― | 78.1 | ― |  |  |  |
|  |  |  |  |  | GZG.V.6574r | 160 | 19.7 | 34.4 | ― |  |  |  |
|  |  |  |  |  | GZG.V.6211r | 195 | 23.7 | 43.2 | ― |  |  |  |
|  |  |  |  |  | GZG.V.6314l | 290 | ― | 68.9 | ― |  |  |  |
|  |  |  |  |  | GPIT/RE/4156r | 85 | ― | 18.7 | 35 |  |  |  |
|  |  |  |  |  | GPIT/RE/3524l | ― | 17.8 | 31.6 | 53 |  |  |  |
|  |  |  |  |  | GPIT/RE/3522r | 128 | 15.3 | 29.1 | 45 |  |  |  |
|  |  |  |  |  | GPIT/RE/3584l | 208 | 25 | 46.1 | 75 |  |  |  |
|  |  |  |  |  | GPIT/RE/3586r | 210 | ― | 45 | ― |  |  |  |
|  |  |  |  |  | GPIT/RE/3580r | 150 | 19.1 | 33.2 | 54 |  |  |  |
|  |  |  |  |  | GPIT/RE/3582r | 206 | 26.8 | 50 | 81 |  |  |  |
|  |  |  |  |  | SMNSoN1l | 308 | 39.8 | 70.8 | 116 |  |  |  |
|  |  |  |  |  | SMNSoN2r | 162 | 19.6 | 36 | 57.5 |  |  |  |
|  |  |  |  |  | SMNS7855l | 211 | 26 | 46.9 | ― |  |  |  |
|  |  |  |  |  | SMNSoN3l | 222 | 28.8 | 52 | 82 |  |  |  |
|  |  |  |  |  | SMNSoN4l | 279 | 37.9 | 63.3 | 108 |  |  |  |
|  |  |  |  |  | SMNSoN5l | 290 | 43 | 70 | 118 |  |  |  |
|  |  |  |  |  | SMNSoN6l | 275 | 32.9 | 61.8 | 97 |  |  |  |
|  |  |  |  |  | SMNSoN7r | 270 | 33.1 | 61.2 | 97 |  |  |  |
|  |  |  |  |  | SMNSoN8l | 144 | 17.9 | 29.2 | 52 |  |  |  |

Explanation of heading-abbreviations: TL – Total length; PMW – Proximal maximum width; MWDC –Mediolateral width at the deltopectoral crest; DMW – Distal maximum width; MTLS – Maximum thickness at lateral shelf; CM – Midshaft circumference. The suffix “l” means left and the suffix “r” means right. All data in mm.
