# Supplementary material for: Bone Histology in Dysalotosaurus lettowvorbecki (Ornithischia: Iguanodontia) – Variation, Growth, and Implications
Source: PLoS One. 2012 Jan 6;7(1):e29958. doi: 10.1371/journal.pone.0029958 (PMC3253128; doi:10.1371/journal.pone.0029958)
Supplement: Text S1 — This text comprises a more comprehensive description of the thin sections of all five skeletal elements of Dysalotosaurus and additionally includes notes on the modes of preservation of the bone microstructure as well as on the occurrence and distribution of osteocyte lacunae and Sharpey's fibers. (DOC) [file pone.0029958.s003.doc]

Results

The preservation of thin sections is variable and includes extraordinarily good preservation as well as strongly obscured sections with numerous cracks and even completely lost parts. Most of the obtained thin sections show numerous diagenetic fractures, ranging from simple thin lines to wide, crystallized fractures, which disturb the original shape of the cross section. The bone tissue is often altered by diagenetic recrystallisation or infilling by dark to opaque metal oxides. There are also samples in which the bone tissue has conspicuous net structure that obscures most of the original vascular pattern (Figure S1). It is suggested that parts of the mineral content were dissolved in this area during diagenesis.

Dissolution and recrystallisation occur mainly in strongly broken samples and nearby strongly fractured parts. Dark colored metal oxides may obscure most of the bone walls, and this occurs either independently of structure or the oxides are strongly concentrated along and inside of vascular canals. There are also occasional changes in color, which sometimes run parallel to the outer edge of fracture surfaces and may be the result of penetrating fluids circulating within the ambient sediments. The marrow cavities are always filled with fine marl, large crystals or both.

1. Bone Histology of the Femur of *Dysalotosaurus*

The femoral cross section is generally triangular in shape and slightly wider lateromedially than anteroposteriorly. The section becomes more slender proximally, close to the base of the fourth trochanter, and the anteroposterior length here is greater than the lateromedial length (see Fig. 1A-D). The general orientation of the sections is roughly constant, although most of the units are slightly displaced anti-clockwise (in right elements) in more proximal sections. One sharp corner is always directed anteriorly and the other two posterolaterally and posteromedially (in proximal sections more laterally and posteriorly, respectively). The three thinner bone walls are located posteriorly (posterolaterally), anteromedially (medially), and anterolaterally. The thickest part of the compact bone wall is mostly the posteromedial corner, whereas the thinnest part is found either in the posterior or anterolateral wall (Tab. 1).

The edge of the marrow cavity is well defined and mainly consistent. Undulations and pseudo-cavities only occur internal to compact coarse cancellous bone tissue (CCCB). No spongiosa were observed within the marrow cavity. A layer of endosteally deposited lamellar bone is developed only inconsistently and with variable thicknesses around the marrow cavity. This layer never forms a completely surrounding band, but occurs in isolated segments, which are mainly located internal to the CCCB anteriorly and laterally. It may also occur sporadically medially as well as internal to fibrolamellar primary bone, and it is more abundant in larger cross sections.

The marrow cavity has resorbed parts of the compact bone wall mainly posteromedially, posteriorly, and posterolaterally, which is approximately opposite to the location of CCCB. Possible growth cycles and bone laminae are partially resorbed as well, so that they disappear at an acute-angle into the marrow cavity on either side of the posterior bone wall (e.g. Fig. 1B, D; 2A, B).

The compact bone wall consists mainly of two types of bone tissue. Most of it is occupied by fibrolamellar bone tissue with woven fibered matrix and numerous primary osteons (Fig. 2A-D). Only large parts of the anterior corner also consist sometimes of strongly birefringent parallel-fibered matrix (Fig. 2E-F). Compacted coarse cancellous bone (CCCB) is embedded between the fibrolamellar tissue and the marrow cavity. It is mostly restricted to the anterior corner and adjacent areas, but it extends from its anterior position mainly along the lateral side of the cross section (Fig. 1B, D; 2G-H . In more distal sections, the amount of CCCB relative to fibrolamellar bone increases and the anterior corner may even be formed entirely by this tissue. Distally within the shaft, the average size of the innermost canals of the CCCB also increases due to the less advanced lamellar infilling.

The vascularization (*sensu lato*, following [6]) is very variable in terms of the size of the canals and overall density. Generally, most of the vascular canals are well developed primary osteons. Their highest density is observed in the posteromedial corner and adjacent areas (Fig. 3A-B). Here, they are also relatively large and there is only a small amount of bone matrix in between them. Especially in the anterior corner and lateral wall, the relative amount of bone matrix is much higher and the density of mainly circumferential primary osteons is lower (Fig. 3C-D). The smallest, longitudinal, and fairly well-organized primary osteons are observable in the innermost areas anterolaterally, anteriorly, and anteromedially close to the CCCB. There are relatively thick cords of matrix, which isolate these osteons from one another and resemble a knitted pattern (Fig. 3E-H).

The organization pattern of vascular canals is relatively variable, but the laminar type is most abundant. Other patterns are observable depending on the particular area in the cross section. As a primary tendency, the organization of vascular canals is high in the thin walls and low in the posterolateral and posteromedial corners (compare Figs. 3A-B with 3C-D). The anterior corner is a special case, because the actual periosteal compact bone wall is often much thinner than the other units due to the internally deposited wedge of CCCB (Fig. 3E-F). Thus, there is an especially high degree of organization (Figs. 2E-F; 3E-F). Another tendency is the general increase of vascular organization from the perimedullary region towards the external surface. In large cross sections, the internal regions reveal a mixture of longitudinal (close to the anterior corner), laminar (also anterior and as part of slow growing zones), plexiform, and even reticular patterns. The laminar type becomes more dominant towards the outer surface until it is the only type left (in large sections). Plexiform and reticular patterns occur mainly in the posteromedial and posterolateral corners. The area within and adjacent to the posterolateral corner is dominated by reticular vascularization.

Moreover, this corner represents a special area within the bone wall (Fig. 4A-B). Here, vascular canals and rather weakly developed primary osteons are larger on average and more randomly shaped and orientated (Fig. 4C-D). Overall, this cluster of canals ascends slightly at the edge of the marrow cavity laterally, and then becomes very steep close to the posterolateral corner, where it finally reaches the external surface (Fig. 4B). Many of the vascular canals are even radially oriented in this direction. This area, which will be called Posterolateral Plug in the following text, represents a very abrupt change within the organization of bone tissue. The general course of the posterior growth cycles, bone laminae, and the orientation of vascular canals stops at the border of the Posterolateral Plug (Fig. 4B) and only distinct annuli/LAGs can be followed through it. This area is most prominent in sections slightly distal to the midshaft and becomes less prominent proximally towards the fourth trochanter.

In sections at the base of the fourth trochanter, the Posterolateral Plug as well as the posterolateral corner itself is very weak and the posteromedial corner is instead much more pronounced and pointed. However, a structure similar to the posterolateral corner is not developed. Such a structure is sometimes visible in the outer cortex of the anterior corner in larger and more proximal sections (Fig. 4E-H). This cluster does not significantly disturb the general organization of the tissue and is also far less widespread.

The zonation pattern is highly variable. LAGs and/or annuli are present (Fig. 5A-B), but only in 10 out of 30 sampled femora. There is additionally no correlation between the size of the bone and the number of LAGs and annuli, so there are medium sized femora with a LAG and large femora without one (Fig. 1B, D). None of the cross sections record more than one or two LAGs and/or annuli. Nevertheless, these are the only growth cycles that can be followed around the cross section.

Another type of growth cycle is much more abundant, but less distinctive than annuli/LAGs because it is often only clearly visible under polarized light (Fig. 5C-D). It is very difficult to follow these zones for even as little as half of the cross sectional circumference. In most cases, these zones are most developed within the lateral side of the posterior wall close to the Posterolateral Plug (Figs. 1A-D; 5E). This kind of growth cycle includes weakly birefringent (under polarized light) fast growing zones with mainly longitudinally oriented collagen fibrils as well as numerous and dense primary osteons showing a relatively lesser degree of organization (Fig. 5F-G). The fast growing zones alternate with more strongly birefringent slow growing zones, which consist of mainly transversely oriented collagen fibrils and less dense and more circumferentially orientated primary osteons showing a relatively higher degree of organization (Fig. 5F-H). The relative distances between primary osteons are larger than in the fast growing zones. The transition from the fast growing to the slow growing zone is diffuse. In contrast, the external rim of the slow growing zones is the best definable part of the whole growth cycle and LAGs and/or annuli mainly occur in this area. In summary, one growth cycle consists of an internal fast growing zone and an external slow growing zone.

Within the anterior corner, by contrast, the slow growing zones often merge together to form a relatively uniform slow growing area occupying the whole thickness of the bone wall. The slow growing zones can also split up. Splitting occurs mainly in the transitional area from a thinner wall to a thicker corner. Inside the very thick posteromedial corner, the slow growing zones become indistinct or can even vanish and only LAGs or annuli remain. The Posterolateral Plug interrupts the course of growth cycles completely (Figs. 4A; 5C-E).

In five of the largest sectioned femora, there is a transition (Mark of Initial Sexual Maturity – MISM) between the generally distinct sequence of growth cycles internally and a much more uniform area externally. The latter resembles a very thick slow growing zone, although a weak internal zonation is still recognizable (Figs. 1A-B; 5B; 6A-E). In a sixth specimen, there is no such mark and the posterior wall bears a normal succession of growth cycles throughout its entire thickness.

Secondary remodeling is very rare. There are only local occurrences of scattered secondary osteons, concentrated mainly in the inner part of the anterior corner along the border between the primary bone tissue and the wedge of CCCB (Fig. 6F-H). Isolated osteons are also present within the CCCB (Fig. 2G-H). Other isolated occurrences are located within the Posterolateral Plug (Fig. 4C-D), where clusters of scattered or several isolated secondary osteons are sometimes observable at variable distances from the external surface. Even rarer are isolated secondary osteons in the outer part of the anterior corner in more proximal sections (Fig. 2E-F). In more distal cross sections, and closer to the metaphysis, secondary osteons become more abundant, especially in the remaining areas of endochondral tissue, but there are never numerous generations establishing a Haversian system. However, sections from more distal parts of the femur shaft have greater numbers of secondary osteons (as in a *Plateosaurus* fibula; [26:56]).

Osteocyte lacunae are generally very abundant, although there are quite large differences in density. The highest densities occur around the border between CCCB and the primary bone wall anteriorly and laterally, and within the Posterolateral Plug (Fig. 4D). The density of osteocyte lacunae is always higher in areas where secondary remodeling is active (Figs. 2E-F; 4C-D). There are often differences between the two main parts of each growth cycle and between different main units of a cross section. The fast growing zones possess relatively more osteocyte lacunae than the slow growing zones. The lacunae of the fast growing zones are much more rounded than the often flattened lacunae in the slow growing zones (Fig. 5G-H). The anterior corner and the walls of the cross sections possess a relatively lower density of osteocyte lacunae than the posteromedial and posterolateral corner and adjacent areas.

Sharpey’s fibers are relatively common, but mainly as isolated and scattered bundles of variable extent. Many are only visible by polarized reflection of their angled direction towards the external surface. Others have the appearance of a swarm of isolated black fibers under normal light (e.g. Fig. 5B). Some occur deep within the cortex and others close to or at the external bone surface. Although the size, orientation, and abundance are highly variable between different cross sections and even within a single section, there is a general repeated pattern. The medial side of the anterior corner often possesses fiber bundles directed medially. Further medially along the medial wall towards the posteromedial corner, the fibers become directed more posteromedially and posteriorly. The posterior wall sometimes possesses posteriorly directed Sharpey’s fibers. More complicated is the arrangement in the lateral side of the cross sections. Here, fiber bundles with posterior to posterolateral direction occur directly within and adjacent to the Posterolateral Plug, but soon after reaching the lateral wall, there may be fiber bundles with an anterolateral direction. The posterolaterally and anterolaterally directed fiber bundles can even cross each other along most of the lateral wall. Finally, there are sometimes small anteriorly directed fiber bundles close to the lateral side of the anterior corner.

Longitudinal sections of a large femur and of the smallest sampled femur (Fig. 7) reveal the structure of the distal epiphyseal ends of these bones. The large longitudinal section is thoroughly built by a meshwork of endochondral trabecular bone. The main direction of the bony straps is perpendicular to the plane of the preserved articular end and they are mainly parallel to one another with numerous transverse connections. Close to the marginal rim of the epiphysis, this regular arrangement fades into mainly randomly oriented straps. The individual thickness and length of the straps as well as the density and degree of interconnection of the trabecular meshwork decrease distinctly in this area. The meshwork of bony straps reaches the preserved distal end of the epiphysis, but pads of calcified cartilage are still very common and can extend up to 35µm from the distal rim into the epiphysis (Fig. 7A-C).

In the small femur, the pads of calcified cartilage extend up to 1mm into the epiphysis and isolated pads are also visible randomly between the straps of bone inside the epiphysis. The bony straps themselves are much thinner than in the large femur and the meshwork is much less well developed with larger distances between the straps and less common interconnections. There is, however, a concentration of bony straps in the epiphyseal centre, which reaches almost to the distal end. The ossification at the periphery of the epiphyses is, in contrast, very poor.

2. Bone Histology of the Tibia of *Dysalotosaurus*

CT-scans have revealed that the thickest periosteal cortex is at approximately 30% of the shaft’s length, so most of the sections are located in the distal third of the bone. As in the femur, the cross section can be subdivided into units that help to clarify its original position in the articulated bone (Fig. 1E-H). The anterior wall is very straight, almost parallel to the mediolateral axis of the bone, and represents the articular surface for the fibula. The anterolateral corner is often the thickest part of the cross section. The anteromedial corner is less acute, but possible growth cycles change their course abruptly. The rest of the section consists of a relatively consistently curved posterior arch. This egg-like shape (Fig. 1G-H) becomes more circular proximally (Fig. 1E-F), but the straight anterior wall persists. The thinnest parts are either found in the anterior wall or in the lateral part of the posterior arch (Tab.2).

The shape of the marrow cavity is more symmetrical than the external outline and its outer edge is mostly well defined and straight, except in some cases internal to the anterolateral corner. A slight shift of the marrow cavity medially occurs during ontogeny.

The endosteal layer is developed almost exclusively in medium to large sections (Fig. 9A-B) with its maximum thickness in the anteromedial or anterolateral corner. There is, however, only one example in which the endosteal layer surrounds the marrow cavity completely. In the other sections, long segments of this layer may be widely separated from one another.

As in the femora, the tibia cross sections consist generally of fibrolamellar bone tissue of fibrolamellar bone tissue with a high density of well-developed primary osteons, which are predominantly organized in a laminar pattern (Fig. 9A-B). The other type of tissue is CCCB, which can occur as a wedge in the anterolateral corner internally. It extends far into the cortex only in the two largest cross sections (Figs. 1G-H; 9E). In most of the smaller sections and in the proximal ones, CCCB is absent.

The development, density, and organization of primary osteons is, also similar to femora, strongly dependent on the respective area within the cross section. The medial part of the bone wall, and especially the anteromedial corner, is very densely packed with often relatively large primary osteons and only small amounts of bone matrix in between (Fig. 9C). Generally, the innermost areas of the medial and posterior units, and sometimes also anterolaterally close to the border of the CCCB, consist of small, laminar organized, longitudinal osteons with relatively weak lamellar infilling (knitted pattern; Fig. 9A-B, G). The density of osteons also decreases slightly close to the external surface and the canals are often still open at the periphery even in the largest sectioned specimen. Less filled, but mainly larger and randomly orientated osteons are found anterolaterally approximately in the middle of the cortex. This roughly circular structure is very similar in its appearance to the Posterolateral Plug of the femora and also interrupts the zonation pattern (Fig. 9E, H). However, its extent within the shaft is much smaller, because it vanishes almost completely in slightly more proximal cross sections.

The vascularization pattern is, as in femora, dominated by laminar organization (Fig. 9A-B), although there is also a high variability. Generally, the thinner the bone wall of a cross sectional unit the higher is the degree of organization. The degree of organization also increases towards the external surface and within slow growing zones. Thus, the laminar pattern dominates mainly in the anterior and posterior to posterolateral walls, in slow growing zones, and in the outer cortex (Fig. 9D), whereas plexiform and reticular patterns occur in the anteromedial (Fig. 9C), medial, and anterolateral bone walls, in the fast growing zones, and in the inner cortex. Reticular patterns are mainly developed in the Anterolateral Plug (Fig. 9H). The mainly longitudinal arrangement is, as described above, only preserved in the innermost parts of some cross sectional units.

The zonation pattern is similar to that of the femora, consisting mainly growth cycles with fast and slow growing zones and very few LAG’s or annuli (Figs. 1F, H; 9A, E; 10; Tab. 2). There is also splitting and merging of slow growing zones and the number and arrangement of these zones is variable between cross sections. In most sections, the distance between slow growing zones decreases towards the anterolateral corner and increases towards the entire medial side (Fig. 1H). This discrepancy of distances vanishes in more proximal sections (Fig. 1F). The growth cycles are best preserved in the anterior and/or medial wall (Fig. 10C-F) and, in contrast to LAGs and annuli, they are not traceable around the bone wall. A transition from a distinct pattern of growth cycles internally to a more uniform area externally, as occurs in five large femora, is not present, although only two large tibiae could be sampled.

Secondary remodeling is much scarcer than in femora. The only unit with preserved secondary osteons is the anterolateral corner. Scattered examples are found mainly in the outer area of the CCCB wedge and within the Anterolateral Plug (Fig. 9F, H). Small sections or sections from more proximal levels lack secondary osteons completely.

In one of the large tibial cross sections (SMNS T3), at the anterior edge of the marrow cavity, an unusual bone tissue is preserved (Figs. 1G-H; 11). It is strongly cancellous with irregularly shaped cavities of various sizes. It is weakly birefringent under polarized light. It is also clearly separated from the compact bone wall by an endosteal layer (Fig. 11C-D, G-H). Some of this tissue was also found inside two large cavities within the CCCB-wedge (Fig. 11A-D). All these features indicate that this tissue belongs to the endosteal type of tissue called medullary bone.

As in the femora, the osteocyte lacunae are more abundant in the fast growing zones than in the slow growing zones. The highest density occurs in the Anterolateral Plug and in areas of secondary remodeling including the external rim of the CCCB wedge (Fig. 9G-H). The density generally decreases from the inner to the outer areas in all units.

Sharpey’s fibers are relatively rare throughout the tibial cross sections, except in the anterolateral corner. Generally, the fiber bundles are more or less anterolaterally directed at the medial side of this corner and anteriorly directed at its lateral side. If the Anterolateral Plug is present, many more directions are possible and fiber bundles are also present in the middle cortex, which is often only visible under polarized light (Fig. 9E). A few weak anterolaterally directed fiber bundles are known from the anterior wall, anteriorly directed bundles from the anteromedial corner, anteromedially directed bundles from the medial wall, and laterally to posterolaterally directed bundles from the lateral wall. Most of these occur only in some of the sections.

3. Bone Histology of the Humerus of *Dysalotosaurus*

The shape of the cross sections varies from a lateromedially wide and flat oval outline distally to an almost circular oval shape more proximally (Fig. 1I-L). However, the shape and relative location of the marrow cavity, the relation of bone wall thickness to the size of the marrow cavity, and the arrangement of growth cycles helped to estimate the relative position of the section in the shaft of the respective humerus. Thus, cross sections taken distal to the mid diaphysis have relatively wide or large marrow cavities compared to the overall very consistent bone wall thickness (Figs. 1I-J; 13E). The cavity is also in a central position of the cross section and has a consistently and lateromedially wider oval shape. All types of growth cycles are arranged in a very consistent pattern, where the distances between one another as well as the distances to the external and internal edge of the bone wall do not vary significantly (Fig. 13E-F). In contrast, the external outline and the shape of the marrow cavity of the single section taken at or near the mid diaphysis (GPIT/RE/4262) are nearly circular. The bone wall is much thicker relatively to the marrow cavity and the latter lies exactly in the cross sectional centre. Nevertheless, the single preserved LAG is quite asymmetrical in its course, because it is resorbed by the marrow cavity medially and it rises up to the outer periphery laterally. Finally, the cross sections taken proximal to the mid diaphysis have again a larger marrow cavity compared to the bone wall thickness than the mid diaphyseal section, but this difference is strongly dependent on the respective distance from the midshaft (Fig. 1K-L). In contrast to the other two cutting levels, the marrow cavity is positioned slightly medial to the cross sectional centre and it is also asymmetrical in shape (Fig. 13A). It tapers slightly laterally because of an internally protruding low bulge of the anterolateral bone wall. Finally, the course of growth cycles is also asymmetrical, so that the inner ones may be resorbed by the marrow cavity medially and are much closer to the external surface laterally. The distance between the growth cycles is also much smaller laterally than medially (Fig. 1K-L).

CCCB is very rare and only visible in the most distal sections and in the anterolateral part in the most proximal sections. More common is the preservation of an endosteal layer, although it never surrounds the marrow cavity completely. Sections slightly proximal to the middle of the shaft possess an endosteal layer of various thicknesses along the lateral rim of the marrow cavity (Fig. 1K-L) or there is a thick but short wedge concentrated in the anterolateral corner of the cavity (Fig. 13A-B).

The bone matrix of the primary compact bone wall consists mainly of fibrolamellar bone tissue, although the anterolateral corner may be formed by brightly reflecting bone tissue of almost parallel-fibered type in some of the more proximal sections (Fig. 13C), which is similar to the anterior corner of proximal femoral sections. However, this Anterolateral Plug is only visible in mid diaphyseal and proximal sections and is also much less distinct than in femora and tibiae.

Primary osteons are numerous and dense, but there are high numbers of relatively smaller and longitudinal osteons with a strongly birefringent single ring of lamellar infilling (Fig. 13D). Such small primary osteons are absent in femora and tibiae. The usual, well developed type of osteons is more common in the medial side of the humeral cross sections and within distinct fast growing zones, but both osteonal types may occur together. The inner parts, as well as distinct slow growing zones, often have less well-developed and/or smaller primary osteons.

The vascularization pattern is laminar dominated, but the organizational degree increases towards the external surface, within the thinner walls (especially anterolateral), and within the slow growing zones (Fig. 13B-D, F-H). The innermost part of the anterolateral or lateral unit often consists of longitudinal vascularization. Mainly in proximal sections, there are also large radial canals visible, which can extend throughout the whole thickness of the cortex (Fig. 13G). They are strongly medially inclined, slightly curled up, and become more perpendicular towards the external surface and closer to the medial corner. They are always more distinct and numerous in the posterior wall than in the anterior wall.

LAGs and/or annuli are more abundant than in femora and tibiae, but their distribution is still very inconsistent (Fig. 13E-F, H).

Secondary osteons are very rare. They are often located at the edge of the CCCB in the most distal or proximal sections, but they mainly occur close to the internal margin of the anterolateral corner along the edge of the short endosteal layer (Fig. 13D) or within the Anterolateral Plug, if present.

As in the other long bones, osteocyte lacunae are denser within fast growing zones, in the Anterolateral Plug (Fig. 13C), and in the scarce areas of secondary remodeling. Sharpey’s fibers are common and are similar orientated as the long radial canals with a strong medial inclination, which becomes perpendicular to the external surface in the medial corner itself.

4. Bone Histology of the Fibula of *Dysalotosaurus*

Due to the scarcity of preservation of fibulae, cross sections could only be produced from levels very close to or within their proximal metaphysis. Therefore, periosteal compact bone is, if at all, often present as a thin layer surrounding parts of the bone wall externally and it is impossible to get an accurate count of growth cycles.

The overall shape of the cross sections is oval to kidney-like with very thick and strongly curved bone walls anteriorly and posteriorly, which also represent the long axis of the sections. The lateral unit is consistently convex, whereas the medial wall is distinctly concave. This concavity is bordered anteriorly and posteriorly by medially protruding corners, and these structures combined represent the external attachment site for M. flexor digitorum longus, running from the medial edge of the proximal joint distally.

Most of the outer rim of the marrow cavity (especially in the anterior and posterior corners) is poorly defined because of wide cavernous spaces surrounded by a loose network of trabeculae. A more consistent rim was found along the thinner lateral and medial walls. An endosteal layer is here also present. This band of lamellar bone is usually very thin laterally, much thicker medially, and especially thick posteromedially (Fig. 14 A-D).

The thin layer of periosteal primary compact bone tissue is mainly present at the outermost area of the anterior, lateral, and posterior sides of the cross sections. It consists of fibrolamellar bone tissue, although the primary osteons are often relatively small and not very dense. Most of the thickness of the bone wall consists of already compacted CCCB externally and not yet compacted CCCB internally. Endochondral bone tissue with a dense, diffuse matrix and rare vascular canals is often present between the periosteal bone tissue and the CCCB.

The medial wall differs strongly from the other units, because it is heavily altered by dense Sharpey’s fibers, so that the area is strongly birefringent under polarized light (Fig. 14E-F). The fibers ascend more steeply towards the surface the closer they are to the posteromedial part of the medial wall. The bone matrix seems to be completely metaplastic in origin and the vascular canals are simple, elongated, and orientated parallel to the Sharpey’s fibers.

Secondary osteons are very common in these metaphyseal cross sections. The CCCB is not involved, but its external border and most of the endochondral tissue is, especially in the large specimens, strongly remodeled. Internal and mid cortical areas of the posterior corner may even consist of dense haversian tissue of at least two generations of secondary osteons (Fig. 14 G-H). The medial wall is affected by very coarse remodeling (Fig. 14E-F), because the scattered secondary osteons are rather large. They are also much larger than in the other units and may also affect areas close to the outer surface. The inner part of the medial wall consists mainly of secondary coarse cancellous bone (Fig. 14A-D).

In the cross section of the large fibula GPIT/RE/5109, possible medullary bone is preserved internal to a very thick part of endosteal layer that fans out (Fig. 14A-D). The medullary bone tissue also differs from the CCCB external to the endosteal layer by the lack of birefringent lamellar bone matrix typical for the latter, by the complete lack of any osteonal development, and a much higher density of osteocyte lacunae within its reticular network.

High concentrations of osteocyte lacunae were always found in areas of high activity (secondary remodeling) and assumed high biomechanical stress (medial wall close to the muscle attachment site) (Fig. 14).

5. Bone Histology of the Prepubic Process of the Pubis of *Dysalotosaurus*

In contrast to the other sampled and described skeletal elements, the prepubic process expands almost horizontally and in an anterior direction. Thus, the obtained cross sections are vertically oriented and, in addition to the lateral and medial orientations within the sections, their top and bottom represent now the dorsal and ventral sides, respectively.

Proximal sections, close to the maximum lateromedial width of the prepubic process, have a wide oval shape (Fig. 15A). The external outline is relatively consistent apart from the lateroventral corner, which is slightly acute angled. Sections of more distal/anterior levels from the maximum width of the prepubic process have a triangular to lamp shade-like external outline (Fig. 15B). Here, the lateroventral and medioventral corners are both very acute angled. The dorsal half is deeply but consistently convex, whereas the ventral rim is straight to slightly concave.

The periosteal compact bone wall is very thin compared to the overall diameter of the cross sections. There is no consistent internal margin, because a single large marrow cavity is absent. This space is almost entirely filled with spongiosa. However, some of the internal cavities are quite large, sometimes reaching the relative dimensions of a small marrow cavity. In the proximal sections, the largest of these pseudo-cavities are always located in the medial half of the trabecular area and they become successively smaller towards the lateral side (Fig. 15A). In more distal sections, there are no significant size differences (Fig. 15B). These cavities are always of resorptive origin, because remnants of periosteal compact bone are often still preserved in some of the thicker trabeculae (Fig. 15C-D).

This periosteal compact bone consists of vascular fibrolamellar bone tissue. Well developed primary osteons are mainly visible in the dorsal and medial part of the bone wall, but they are not very dense, mostly longitudinal, and only sometimes slightly plexiform in arrangement (Fig. 15 E). In the ventral bone wall, and sometimes even in parts of the dorsal and medial units, primary osteons are rarer, relatively small, and weakly developed. Here, the matrix is often almost opaque and the mainly simple vascular canals are only longitudinally organized (Fig. 15F). Growth cycles are very rare, but there are at least one to two annuli and/or LAGs preserved in the ventral and/or dorsal unit of the bone wall in some sections.

The entire lateral margin of the bone wall is very different compared to the other units. Instead of woven matrix with vascular canals and/or primary osteons, a highly modified tissue was observed, which is very similar to the medial side of the fibular cross sections (Fig. 14E-F).The parallel-fibered or metaplastic matrix is altered by strong Sharpey’s fibers and many resorptive cavities. The latter are often secondarily filled, so that large secondary osteons are developed. The orientation of these cavities is the same as the lateroventrally directed Sharpey’s fibers.

Numerous small secondary osteons occur in the trabeculae between the large pseudo-cavities as well as in the internal areas of the periosteal compact bone wall, where they sometimes form haversian tissue. Their abundance decreases towards the medial side.

Osteocyte lacunae are, as usual, most dense in areas of secondary remodeling and in areas occupied by numerous Sharpey’s fibers, which is especially observed at the lateral margins of the sections.

Sharpey’s fibers are generally very common and the closer they are located to the lateroventral corner the more they are directed laterally or lateroventrally. Some fiber bundles, those located close to the medial side, are also directed medially and medioventrally.
